# Development of Nanofiber Patch Formulation Containing Rutin Hydrate, In Vitro and In Vivo Evaluation

**DOI:** 10.1021/acsomega.5c01101

**Published:** 2025-06-30

**Authors:** Sinan Özer, Evrim Akyıl, Rana Arslan, Neziha Senem Arı

**Affiliations:** † Faculty of Pharmacy, Department of Pharmaceutical Technology, 52944 Anadolu University, Eskisehir 26470, Turkey; ‡ Faculty of Pharmacy, Department of Pharmacology, Anadolu University, Eskisehir 26470, Turkey; § Faculty of Medicine, Department of Basic Medical Sciences, 552614 Kütahya Health Sciences University, Kütahya 43100, Turkey

## Abstract

Wound healing is a dynamic and multifactorial process that can be significantly impaired by oxidative stress, microbial infection, and chronic systemic conditions, often resulting in delayed recovery and poor tissue regeneration. This study investigates the use of rutin hydrate, a bioactive flavonoid with antioxidant and collagen-promoting effects, in electrospun nanofiber dressings to enhance wound repair outcomes. Electrospinning technology was used to fabricate nanofibers from poly­(vinyl alcohol) (PVA) and Eudragit L100, with optimized parameters determined via the Taguchi method. The nanofibers had average diameters of 258.371 nm (PVA) and 125.115 nm (Eudragit), with drug loading capacities of 78.735 ± 2.307 μg/mg per mass and 87.983 ± 2.055 μg/cm^2^ per area (PVA); 76.833 ± 2.238 μg/mg per mass and 85.807 ± 1.502 μg/cm^2^ per area (Eudragit). Characterization via SEM, FTIR, DSC, and NMR confirmed uniform, bead-free nanofibers with enhanced stability and controlled drug release. In vitro studies showed first-order drug release kinetics (Hixson–Crowell model), balancing burst release with sustained delivery. In vivo wound healing in rats demonstrated significantly faster recovery with rutin-loaded nanofibers (*p* < 0.0001 for F-PVA-Rutin and F-EUD-Rutin on day 3 and day 7). Histological analysis revealed reduced neutrophil infiltration, enhanced granulation tissue, and improved angiogenesis, confirming the therapeutic efficacy of rutin. These findings support the potential of rutin-loaded electrospun nanofiber dressings as an effective and scalable approach for promoting wound healing through localized, sustained drug delivery.

## Graphical abstract

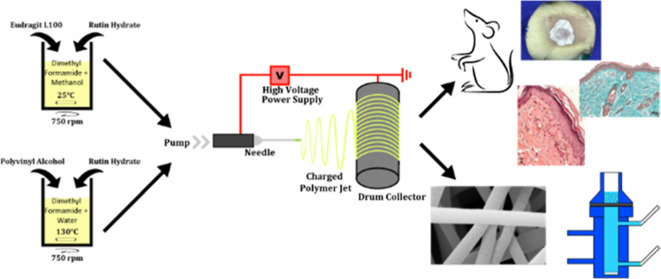

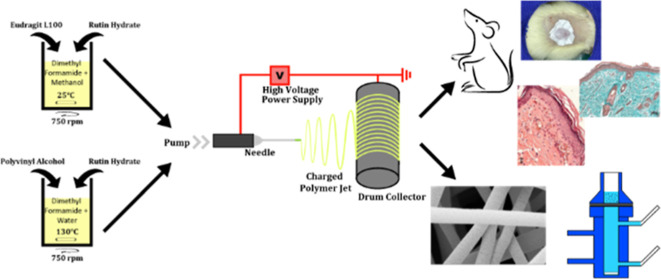

## 1 Introduction

Wounds, defined as disruptions in the integrity of the skin or mucosa, pose a critical challenge in global healthcare, significantly affecting patient quality of life and imposing substantial economic burdens.^1^ The process of wound healing comprises four overlapping phases: hemostasis, inflammation, proliferation, and remodeling.^2^ However, oxidative stress, microbial infections, and chronic conditions frequently impair this process, necessitating advanced therapeutic approaches.^3^

Chronic wounds, affecting approximately 2.5% of the U.S. population, represent a significant medical and economic burden.^4^ The global wound care market, valued at USD 22.25 billion in 2023, is projected to reach USD 29.57 billion by 2030, driven by an increasing prevalence of chronic wounds and demand for innovative therapies.^5^ This highlights the urgent need for advanced wound care technologies that improve patient outcomes and reduce healthcare expenditures.

Among emerging therapeutic solutions, natural compounds such as flavonoids have attracted significant attention for their antioxidant and anti-inflammatory properties, both crucial for wound healing.^6^ Rutin, a flavonoid prevalent in various medical plants, has been shown to promote wound healing by stimulating collagen synthesis and free radical scavenging effects.^7^ However, its therapeutic potential is hindered by poor aqueous solubility and low bioavailability, necessitating innovative delivery systems.^8^

Electrospun nanofiber dressings have gained attention in wound care due to their structural similarity to the natural extracellular matrix (ECM), offering a highly porous and interconnected architecture that supports cell adhesion, tissue regeneration, and controlled drug release.^9,10^ Their flexibility and biocompatibility further enhance their utility across various wound types, including acute, chronic, and burn injuries, by maintaining moisture, reducing microbial risk, and facilitating healing.^11–14^

Wound care poses significant challenges due to the complex interplay of biological and physical factors affecting healing. The development of innovative materials that address these challenges while promoting efficient and cost-effective healing remains a priority. Despite the growing body of literature on nanofiber dressings, few studies have comprehensively evaluated the in vivo efficacy of rutin hydrate-loaded electrospun fibers, particularly using polymers with differing degradation and release profiles such as PVA and Eudragit L100. This study hypothesizes that rutin, a flavonoid known for its potent antioxidant and regenerative properties, can significantly enhance wound healing when incorporated into nanofibers.

The primary research question is Can the combination of rutin hydrate with electrospun nanofibers based on PVA and Eudragit L100 provide a more effective wound healing platform compared to blank and marketed formulations? The primary objective is to design and optimize such nanofiber dressings using either PVA or Eudragit L100 polymers, employing the Taguchi method to achieve controlled drug release and improved mechanical properties. PVA was selected for its biocompatibility, biodegradability, and moisture-retentive properties, making it an ideal polymer for wound healing applications. Eudragit L100, on the other hand, was chosen for its pH-sensitive behavior, which enables controlled drug release in environments where wound pH is elevated, thereby optimizing therapeutic efficacy. By integrating biomaterial engineering with therapeutic innovation, this research aims to provide a scalable and clinically relevant solution to current wound care challenges.

## 2 Materials

Rutin hydrate (≥98% purity, BLD Pharmatech, China), poly­(vinyl alcohol) (PVA, ≥99% hydrolyzed, Sigma-Aldrich, Germany), and Eudragit L100 (Evonik, Germany) were used. The solvents included dimethylformamide (DMF), dimethyl sulfoxide (DMSO), methanol, ethanol, and acetonitrile, all obtained from Merck (Germany). Additional reagents included 2,2-diphenyl-1-picrylhydrazyl (DPPH) and phosphate-buffered saline (PBS) from Sigma-Aldrich (Germany), as well as analytical-grade formic acid.

## 3 Methods

### 3.1 Preparation of Nanofibers

The electrospinning parameters were optimized using the Taguchi method, a systematic approach that identifies critical factors influencing nanofiber characteristics, such as fiber diameter and bead formation. Based on preliminary experiments and a review of relevant literature, four key parameters were selected: polymer concentration, solvent ratio, nozzle-to-collector distance, and needle gauge^15,16^

#### 3.1.1 Preliminary Trials and Parameter Selection

Initial experiments involved varying these parameters to assess their impact on nanofiber formation. Nanofibers containing rutin hydrate were prepared using PVA and Eudragit L100 polymers, dissolved in appropriate solvent mixtures. Fiber formation was first evaluated using light microscopy, and promising samples were further analyzed via scanning electron microscopy (SEM—Carl Zeiss, Germany) for detailed characterization.

The experimental levels of these parameters were optimized to ensure defect-free nanofiber formation. Taguchi experimental designs were created using Minitab 18 software, with detailed experimental patterns summarized in Tables 1 and 2.

**Table 1:** Factors and Levels for Taguchi Design of Nanofibers Prepared with PVA

	factors			
	PVA amount (mg)	water volume in solvent^a^ (mL)	distance (cm)	needle diameter (G)
levels	1050	8	17	17
	1000	7.5	15	19
	950	7	13	21
	900	6,5	11	23

a The total solvent volume consisting of DMF and bidistilled water (BDS) is 13 mL.

**Table 2:** Factors and Levels for Taguchi Design of Nanofibers Prepared with Eudragit L100

	factors			
	Eudragit L100 amount (mg)	DMF volume in solvent^a^ (mL)	distance (cm)	needle diameter (G)
levels	1400	4	11	17
	1350	3.5	13	19
	1300	3	15	21
	1250	2.5	17	23

a The total solvent volume consisting of DMF and methanol (MeOH) is 10 mL.

#### 3.1.2 Solution Preparation

Electrospinning solutions were prepared using two distinct methods: a hot dissolution method^17^ for PVA and a cold dissolution method^18^ for Eudragit L100. Accurately weighed polymers were placed in 40 mL screw-capped glass vials, and the required solvent mixture was added. The solutions were stirred at 750 rpm on a magnetic stirrer (IKA, Germany) using a 20 mm-long magnetic stirrer bar.

For PVA solutions, the stirrer’s heater was set to 140 °C to aid dissolution, while Eudragit L100 solutions were stirred at room temperature without heating. After 22 h of stirring, the PVA solutions were allowed to cool for 1 h before adding rutin hydrate, calculated as 10% of the polymer weight. Stirring was continued for an additional hour to ensure complete dissolution of the active compound. The total preparation time for both solutions was 24 h, after which the mixtures were treated in an ultrasonic bath for 20 min to remove air bubbles and improve solution quality.

#### 3.1.3 Taguchi Experimental Design

A 4-factor, 4-level Taguchi design (4^4^) was employed to systematically evaluate the influence of polymer concentration, solvent ratio, nozzle-to-collector distance, and needle diameter on nanofiber morphology. This design yielded 16 experimental formulations for each polymer (PVA and Eudragit L100), resulting in 32 unique conditions. The component quantities and formulation matrices for both polymer systems are provided in Table S1 and Table S2.

#### 3.1.4 Electrospinning Process

Prepared solutions were loaded into syringes connected to the nozzle via polyurethane tubes, which were placed on a pump. The solutions were advanced toward the nozzle outlet until a droplet formed at the tip. Once the droplet appeared, flow rate and high voltage were applied to initiate the electrospinning process, forming fibers that deposited on the collector. The entire electrospinning process was carried out using a Nanospinner (Inovenso, USA) device.

Key parameters, such as flow rate and voltage, were carefully adjusted to maintain a stable and continuous Taylor Cone, critical for efficient fiber production. The drum collector was configured to move linearly along an 80 mm path at a speed of 3 mm/s, while rotating at 300 rpm around its axis, ensuring uniform fiber deposition.

### 3.2 Solubility Study

The solubility of the active agent was evaluated at room temperature (25 °C) using 10 mL screw-capped glass test tubes as dissolution vessels. Each test tube was loaded with 0.4 g of the active agent, followed by the addition of 5 mL of the solvent. The tubes were tightly sealed with screw caps and wrapped in parafilm to minimize evaporation. The prepared solutions were then placed on a horizontal shaker and agitated at room temperature throughout the experiment.

For each solvent, nine test tubes were prepared, and supernatant samples were collected at 24, 48, and 72 h. At each time point, the supernatants from three test tubes were filtered. To avoid precipitation in saturated solutions, the filtered supernatants were immediately diluted with methanol before analysis.

### 3.3 Characterization of Nanofibers

#### 3.3.1 Morphological Characteristics

The morphological characteristics of the nanofibers, including fiber diameters and bead counts, were analyzed using scanning electron microscopy (SEM—Hitachi, Japan), a standard technique for nanofiber characterization. Imaging was performed under 5 kV high-magnification conditions, employing a charge-reduction observation mode and secondary electron (SE) imaging signals to ensure accurate morphological assessments.

Fiber diameters were measured from SEM images captured at 10,000× magnification, with 15 individual measurements taken for each formulation. The average fiber diameter was then calculated to evaluate the uniformity of the nanofibers. For the optimum formulations, higher-resolution images were acquired at 30,000× magnification using an advanced SEM (Carl Zeiss, Germany), enabling more precise diameter measurements.

Bead counts were assessed from SEM images captured at 10,000× magnification, consistent with established methodologies for evaluating bead density and morphology in nanofiber membranes. The number of beads in the visualized area was manually counted to assess the uniformity and structural quality of the nanofibers.

#### 3.3.2 Spectroscopic Analyses

Spectroscopic analyses of the formulations were performed using Fourier-transform infrared (FTIR—Shimadzu, Japan) spectroscopy and nuclear magnetic resonance (NMR—Bruker Ultrashield CP MAS, Germany) spectroscopy for comparative evaluations.

The chemical compositions and structural characteristics of the formulations were analyzed using FTIR spectroscopy. Infrared spectra were recorded over a wavelength range of 500–4000 cm^–1^, enabling precise identification of molecular vibrations and functional groups within the formulations.

The molecular structures and interactions within the formulations were confirmed using ^1^H NMR spectroscopy. The analyses were conducted with deuterated DMSO (DMSO-*d*_6_) as the solvent, utilizing an Ultrashield CP MAS NMR spectrometer to ensure accurate and detailed molecular characterization.

#### 3.3.3 Thermal Analyses

Thermal properties of the formulations were assessed using differential scanning calorimetry (DSC—Shimadzu, Japan). Approximately 4 mg of each sample was sealed in aluminum pans and subjected to a nitrogen gas flow at 50 mL/min. The samples were analyzed over a temperature range of 50–300 °C with a heating rate of 10 °C/min. This approach enabled the identification of thermal transitions, such as melting points and changes in crystallinity, providing insights into the thermal stability of the formulations.

#### 3.3.4 Mechanical Properties

The mechanical properties of the formulations were evaluated through texture profile analysis (Texture Analyzer—Stable Micro Systems, United Kingdom) . Samples were prepared with dimensions of 3 × 2 cm, oriented with the longer side perpendicular to the applied stress. Testing was initiated when a tensile force of 5.6 g (0.05 N m) was detected and proceeded at a rate of 1 mm/s over an elongation distance of 10 mm. Key parameters such as tensile strength and elongation at break were determined. To ensure the reproducibility and reliability of the results, each test was conducted in triplicate for every formulation.

#### 3.3.5 Antioxidant Activity

The antioxidant activity of the formulations was evaluated using the 1,1-diphenyl-2-picrylhydrazyl (DPPH) radical scavenging assay. A solution containing 25 μg/mL Rutin hydrate in 100 mL methanol was mixed with a 2.5% (v/v) DPPH methanol solution (100 mL). The mixture was incubated in a dark environment for 30 min to prevent light-induced degradation. The absorbance differences were then measured at 517 nm to determine the radical scavenging capacity.

The scavenging activity was calculated using eq 1.^19^ This procedure was also applied to formulations containing Rutin hydrate and to blank formulations (without the active agent) for comparative evaluation.

1 $$Scavenging\;\%=\frac{A_{C}-A_{s}}{A_{c}}$$

Equation 1: Equation for calculating the scavenging activity of formulations, *A*_s_: absorbance of sample *A*_c_: absorbance of control.

#### 3.3.6 Drug Loading

The amount of Rutin hydrate in the nanofibers was quantified using an ultraperformance liquid chromatography (UPLC—Agilent Technologies USA) method equipped with a pump, a photodiode array detector (Max-Light Cartridge Cell—DAD Detector), and an autosampler. Analyses were carried out at a wavelength of 257 nm on a Develosil FlexFire C18 column (100 mm × 2 mm i.d., 1.6 μm), with the column temperature maintained at 40 °C. The mobile phase consisted of a mixture of 1% *o*-phosphoric acid solution, acetonitrile, and methanol in a ratio of 82:16:2 (v/v/v), delivered at a flow rate of 0.4 mL/min. The injection volume was set to 2 μL. Validation studies for the UPLC method were conducted in accordance with ICH guidelines^20^ to ensure the reliability of the results.

To determine the active agent content, a 5 cm^2^ section was randomly selected from the nanofiber sheets, and four 1 cm^2^ samples were taken from the corners and one from the center. The cut samples are shown in Figure S1. The PVA-based nanofibers were dissolved in 1 mL deionized water (BDS), while the Eudragit L100-based nanofibers were dissolved in 1 mL methanol. The resulting solutions were analyzed via UPLC, and the active agent content was quantified as μg per unit area (μg/cm^2^) and μg per unit mass (μg/mg).

#### 3.3.7 In Vitro Drug Release

The in vitro release study of the active agent was conducted using a Franz diffusion cell (Figure 1) system with six cells. Since there is no commercially available wound-healing formulation containing Rutin hydrate, the release profiles of the formulations were compared with that of a pure active agent solution.

**Figure 1:**
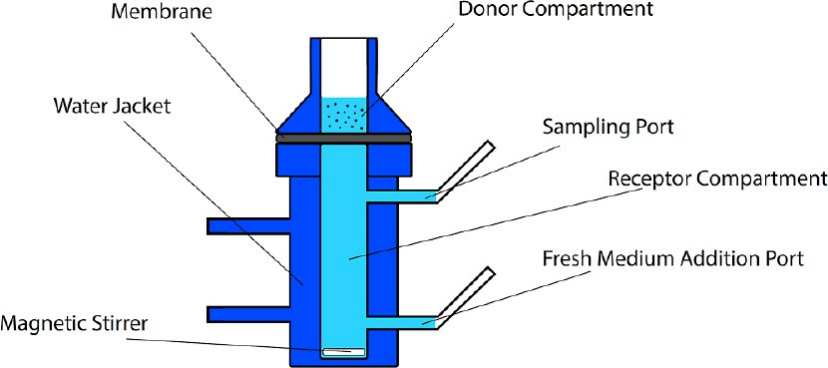
Diagrammatic representation of the Franz diffusion cell (figure created by Dr. Sinan Özer. Copyright 2025).

During the experiment, the receptor compartment was filled with phosphate buffer solution (PBS, pH 7.4) and stirred at 100 rpm. A cellulose membrane filter with a pore size of 0.45 μm was used to separate the donor and receptor compartments. The formulations were cut to match the diameter of the receptor compartment’s surface area and placed onto a preconditioned membrane filter (soaked for 30 min). Since the areas were defined based on the interface between the receptor and donor compartments, minor variations were accounted for by calculating the results as % cumulative release, ensuring that these differences did not affect the final outcomes. Stirring of the receptor compartment at 100 rpm was maintained throughout the experiment using a magnetic stirrer.

For the release study of the pure active agent, an amount of Rutin hydrate equivalent to the average content in the formulations was weighed and applied to the donor side of the membrane, ensuring that the amount used complied with sink condition.

To verify the stability of the active agent under the experimental conditions, a solution of 1000 μg/mL Rutin hydrate in 10 mL of dissolution medium was prepared. This solution was stirred at 100 rpm for 24 h at the same temperature as the release study. The recovery rate (%) of the active agent was calculated to confirm its stability throughout the experiment.

### 3.4 Stability Studies

Stability studies were conducted under three different conditions: 5 ± 3 °C, 25 ± 2 °C (60% RH ± 5% RH), and 40 ± 2 °C (75% RH ± 5% RH). Samples were characterized and compared on the 30th, 60th, and 90th days in accordance with the ICH Guideline.^21^ For stability testing, the nanofibers were placed between two sheets of parchment paper and packaged in aluminum foil to ensure an airtight seal.

### 3.5 In Vivo Efficacy Study

The in vivo efficacy study was conducted on female rats weighing 180–200 g, housed in a well-ventilated room maintained at 22 ± 1 °C with a 12 h light/dark cycle. Animals had free access to food and water, and all care and research protocols adhered to the “Guide for the Care and Use of Laboratory Animals” (NIH Publication no.: 85-23, revised 1985). Our experimental procedure was approved by the Local Ethics Committee of Anadolu University, located in Eskisehir, Turkey, under decision no.: 2020-54, dated December 09, 2020.

In our experiments, excisional wound models were used to investigate full-thickness wound healing. These models offer a standardized and reproducible approach, facilitating the evaluation of therapeutic interventions across species. Modifications like silicone splints enhance their translational relevance, while their application in rodents, rabbits, and pigs allows for controlled wound creation and comparative analysis, making them a valuable tool in preclinical research.^22^

Rats were anesthetized with an intraperitoneal (i.p.) injection of 75 mg/kg ketamine and 15 mg/kg xylazine. The dorsal hair was shaved, and the intended wound area was disinfected with 70% alcohol. Using sterile scissors, a circular wound (12 ± 1 mm in diameter) was created on the dorsal interscapular region, as shown in Figure 12. The wound depth extends through the complete removal of both the epidermis and dermis layers of the skin. Treatments were applied immediately after wound creation according to the groups specified in Figure 13. To ensure unbiased results, only one wound was created per animal, and an untreated control group was included for comparison. Postprocedure, an analgesic dose of 100 mg/kg acetylsalicylic acid was administered intraperitoneally as a one-time application. To avoid potential interactions or injuries among animals, all rats were housed individually in separate cages for the duration of the experiment.

**Figure 2:**
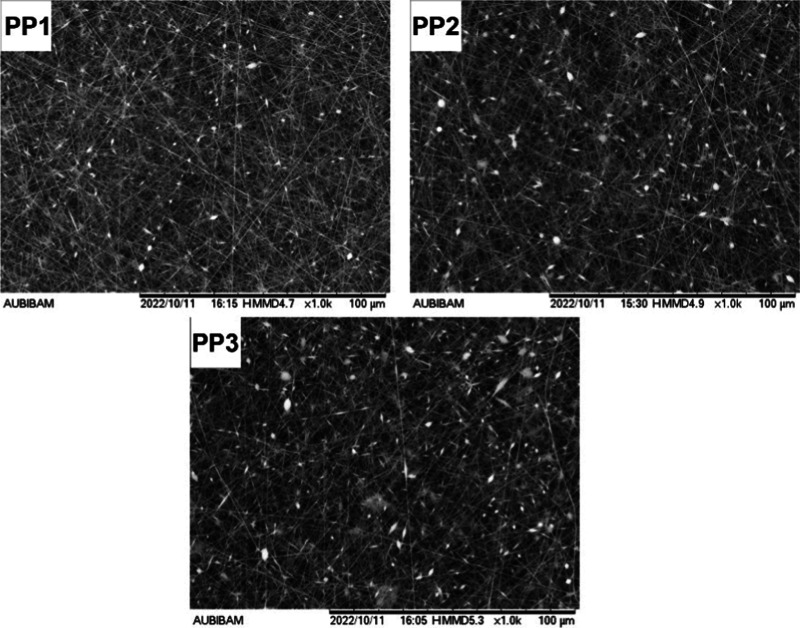
SEM images of nanofibers produced to investigate the effect of PVA concentration on fiber diameter.

**Figure 3:**
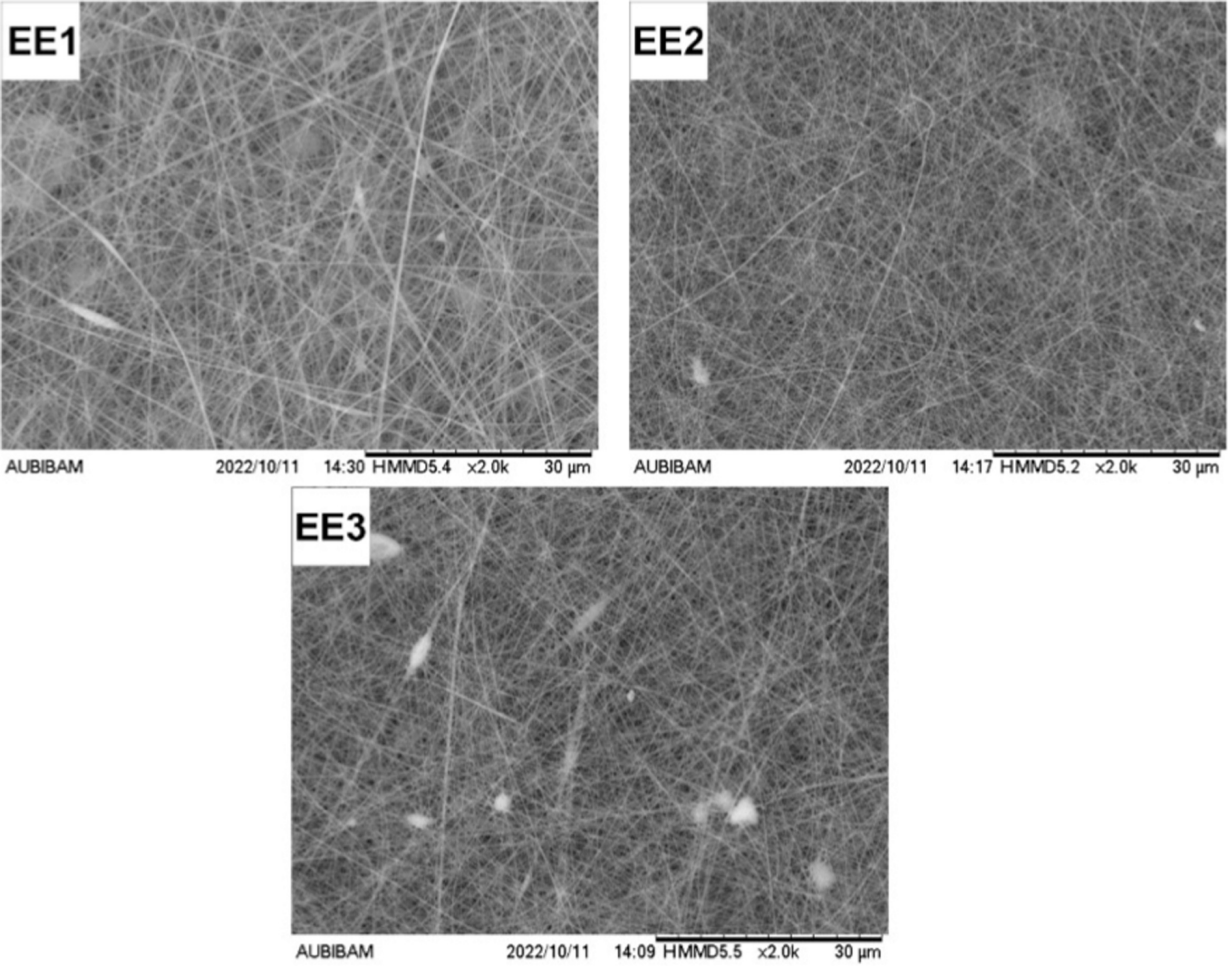
SEM images of nanofibers produced to investigate the effect of Eudragit L100 concentration on fiber diameter.

**Figure 4:**
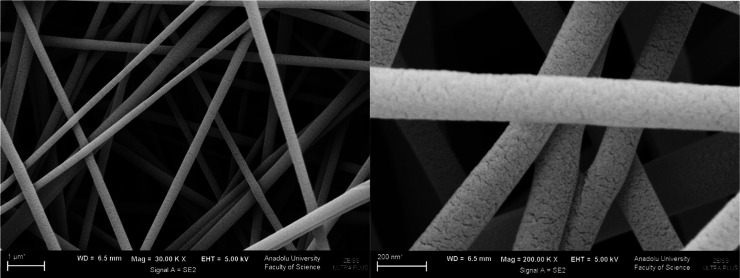
SEM images of the F-PVA-Rutin formulation (Left: 30k× magnification; Right: 200k× magnification).

**Figure 5:**
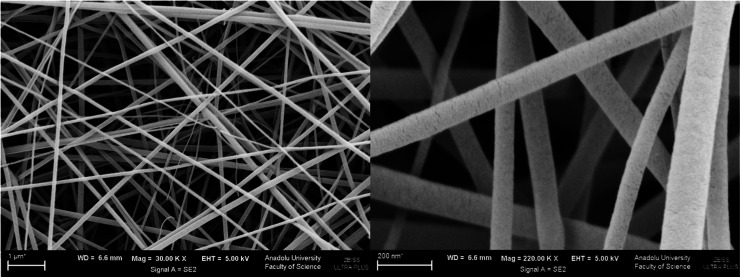
SEM images of the F-EUD-Rutin formulation (Left: 30k× magnification; Right: 200k× magnification).

**Figure 6:**
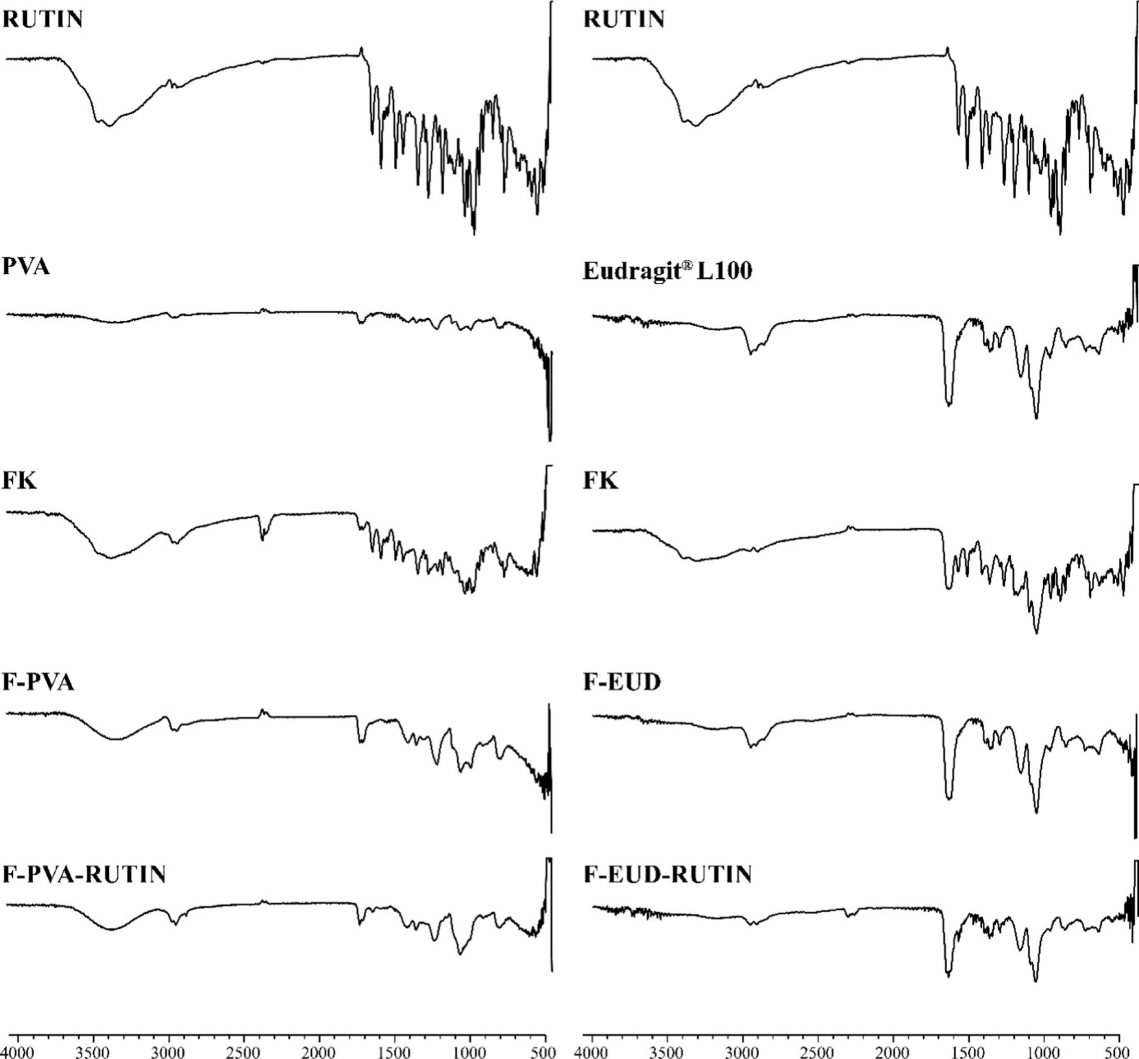
Comparative FTIR spectras (Rutin: Rutin hydrate, PVA and Eudragit L100: pure polymers, FK: physical mixture, F-PVA and F-EUD: drug-free formulations, F-PVA-Rutin and F-EUD-Rutin: drug-loaded formulations.).

**Figure 7:**
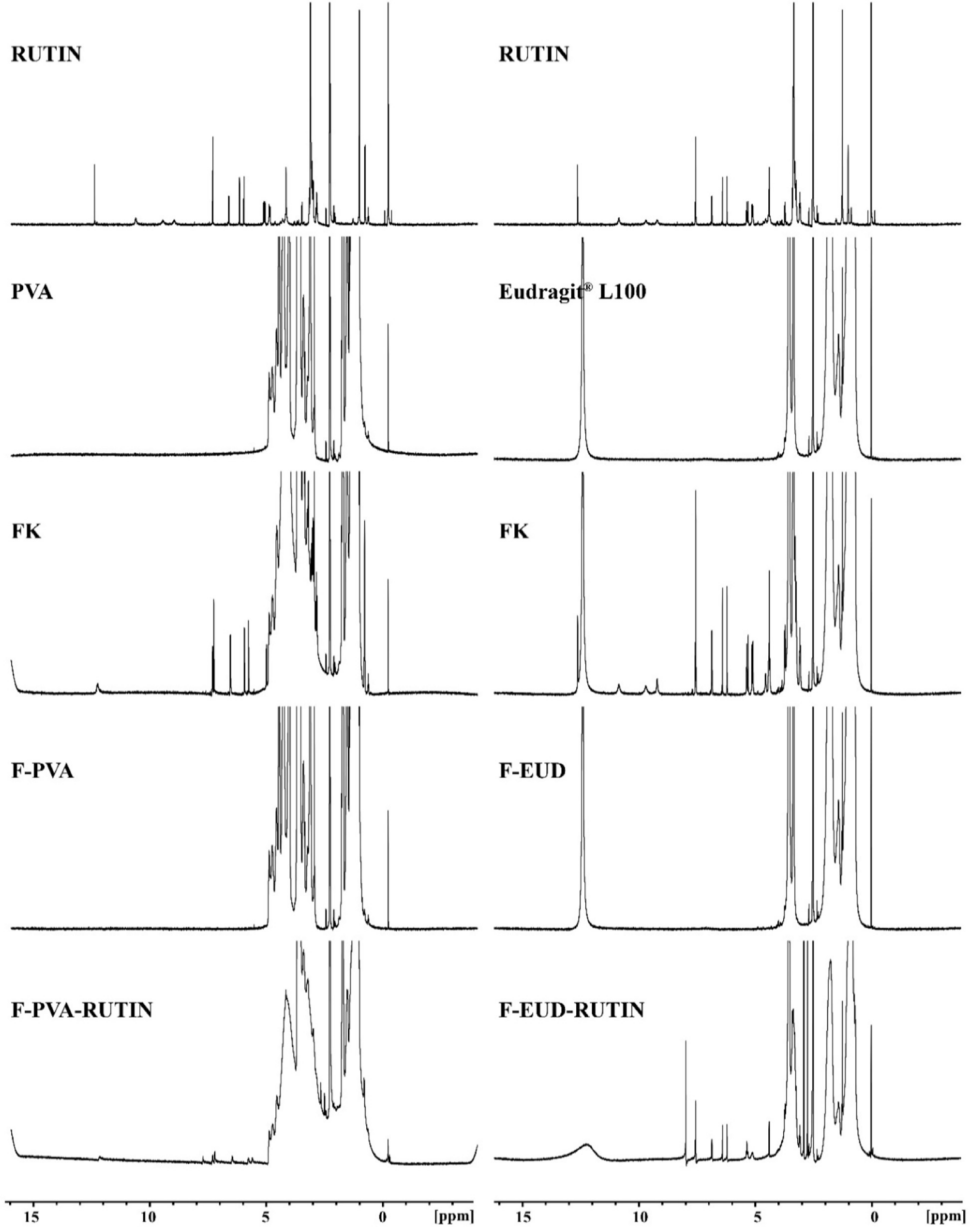
Comparative NMR spectras (Rutin: Rutin hydrate, PVA and Eudragit L100: pure polymers, FK: physical mixture, F-PVA and F-EUD: drug-free formulations, F-PVA-Rutin and F-EUD-Rutin: drug-loaded formulations.).

**Figure 8:**
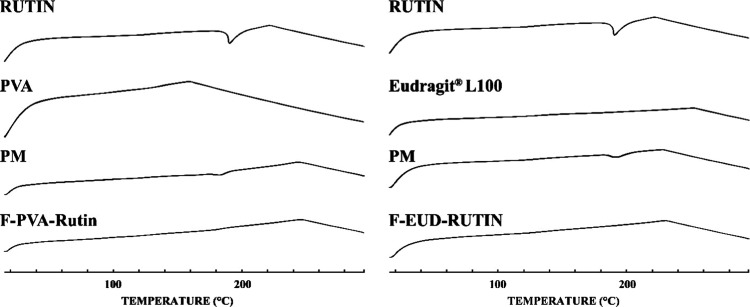
Comparative DSC thermograms (Rutin: Rutin hydrate, PVA and Eudragit L100: pure polymers, PM: physical mixture, F-PVA and F-EUD: drug-free formulations, F-PVA-Rutin and F-EUD-Rutin: drug-loaded formulations.).

**Figure 9:**
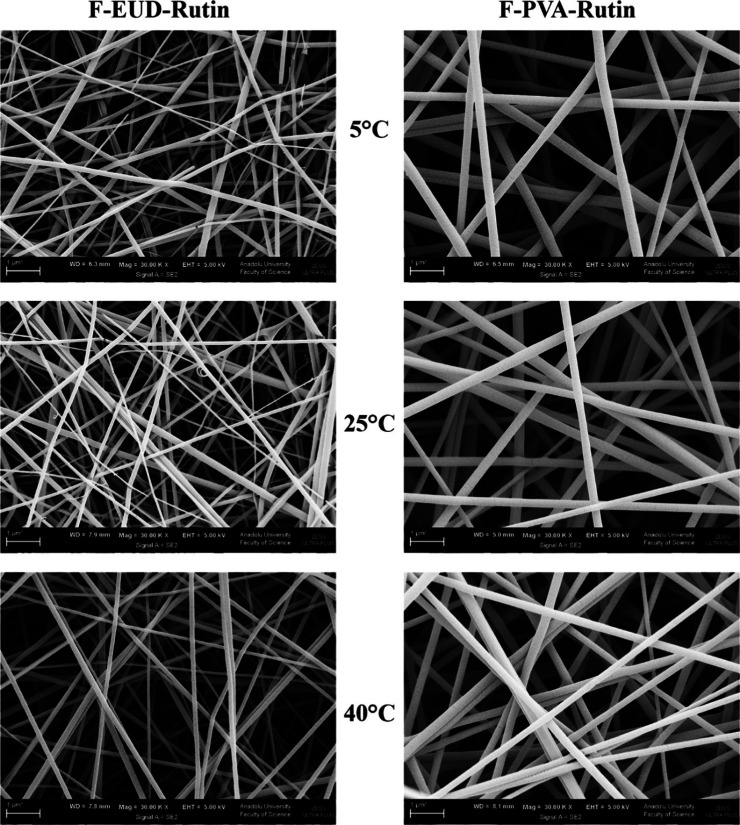
SEM images showing morphological changes in formulations stored at different temperatures on the 90th day of the stability study.

**Figure 10:**
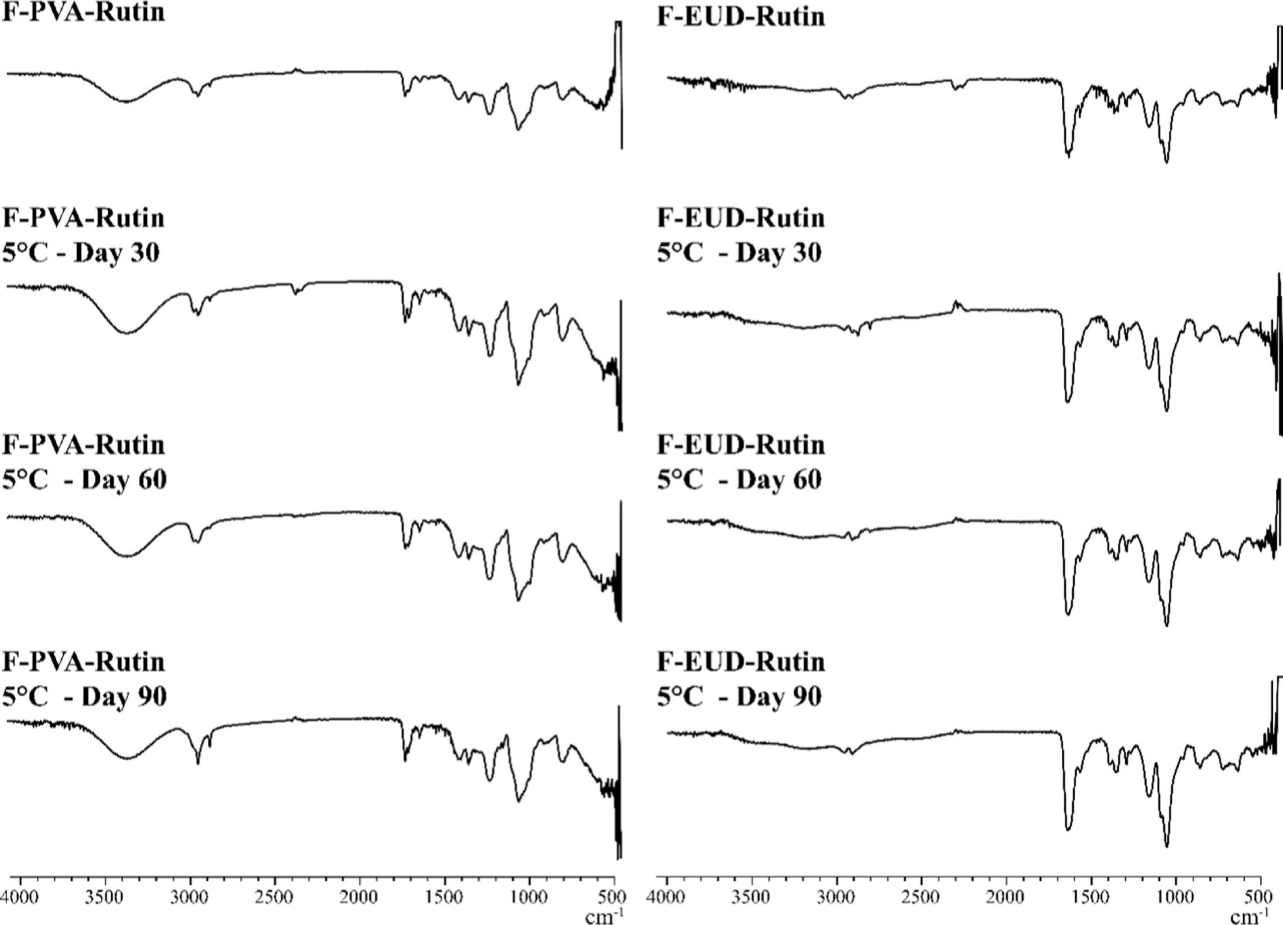
FTIR spectra for stability studies (5 °C).

**11:**
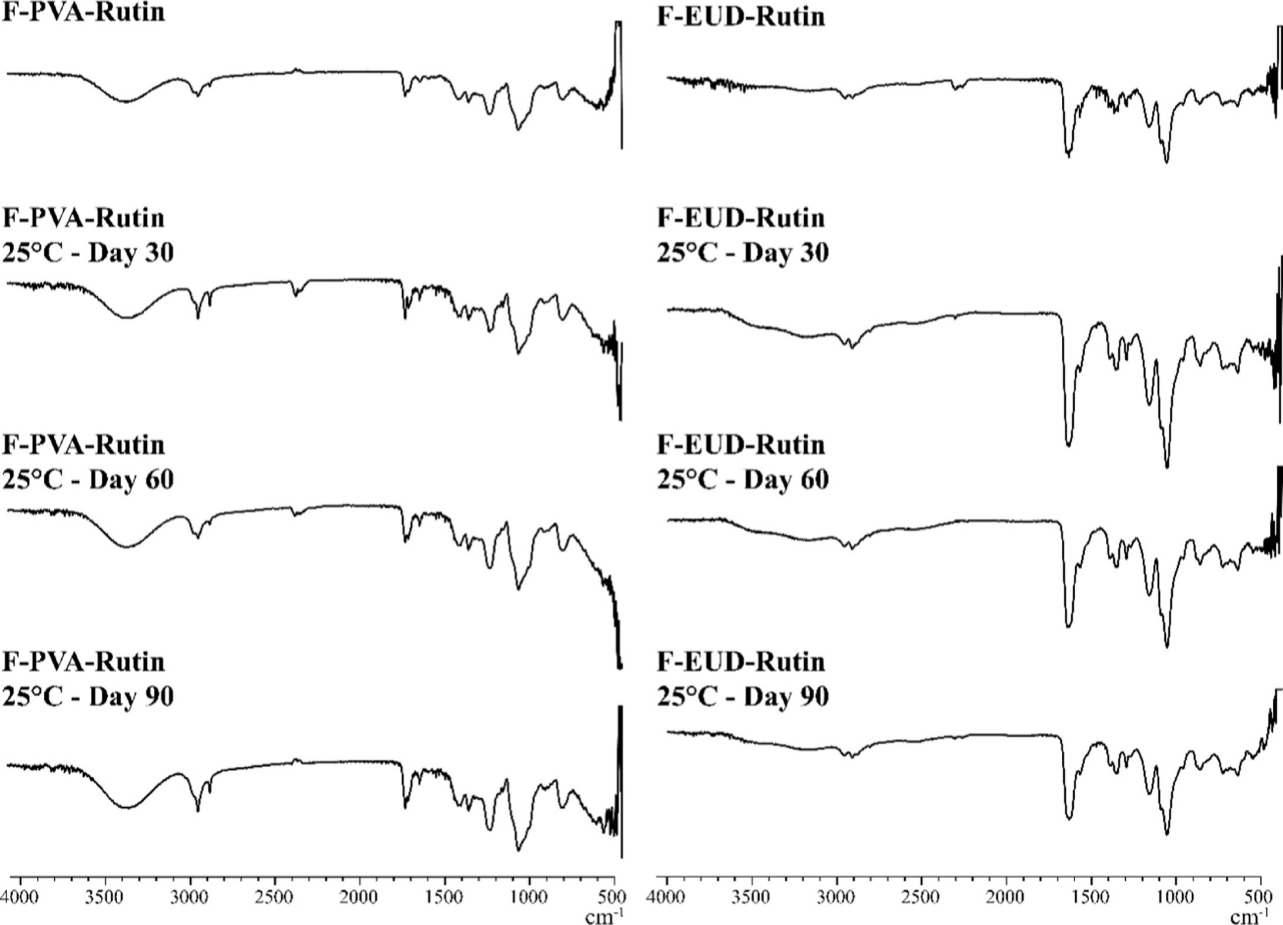
FTIR spectra for stability studies (25 °C).

**Figure 12:**
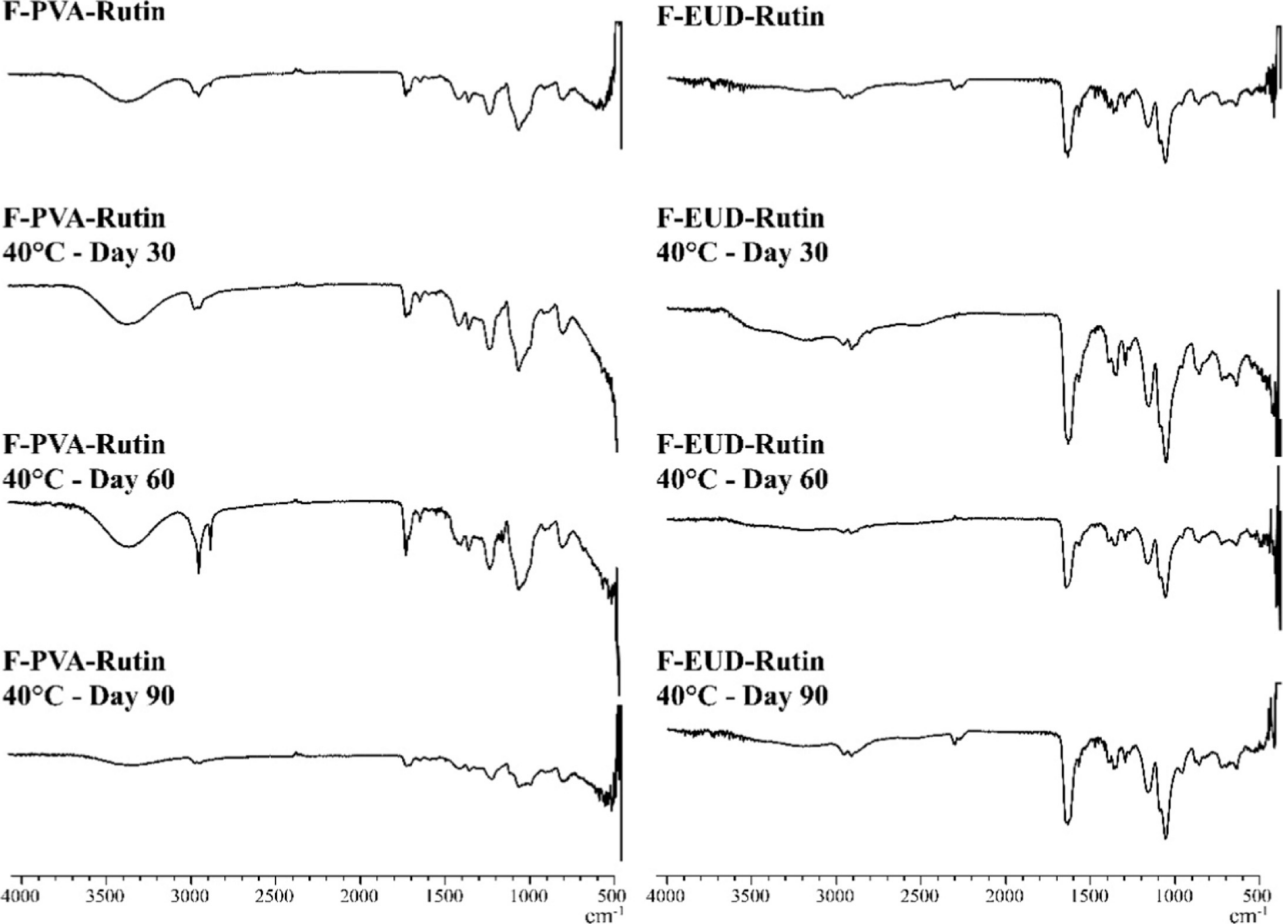
FTIR spectra for stability studies (40 °C).

**Figure 13:**
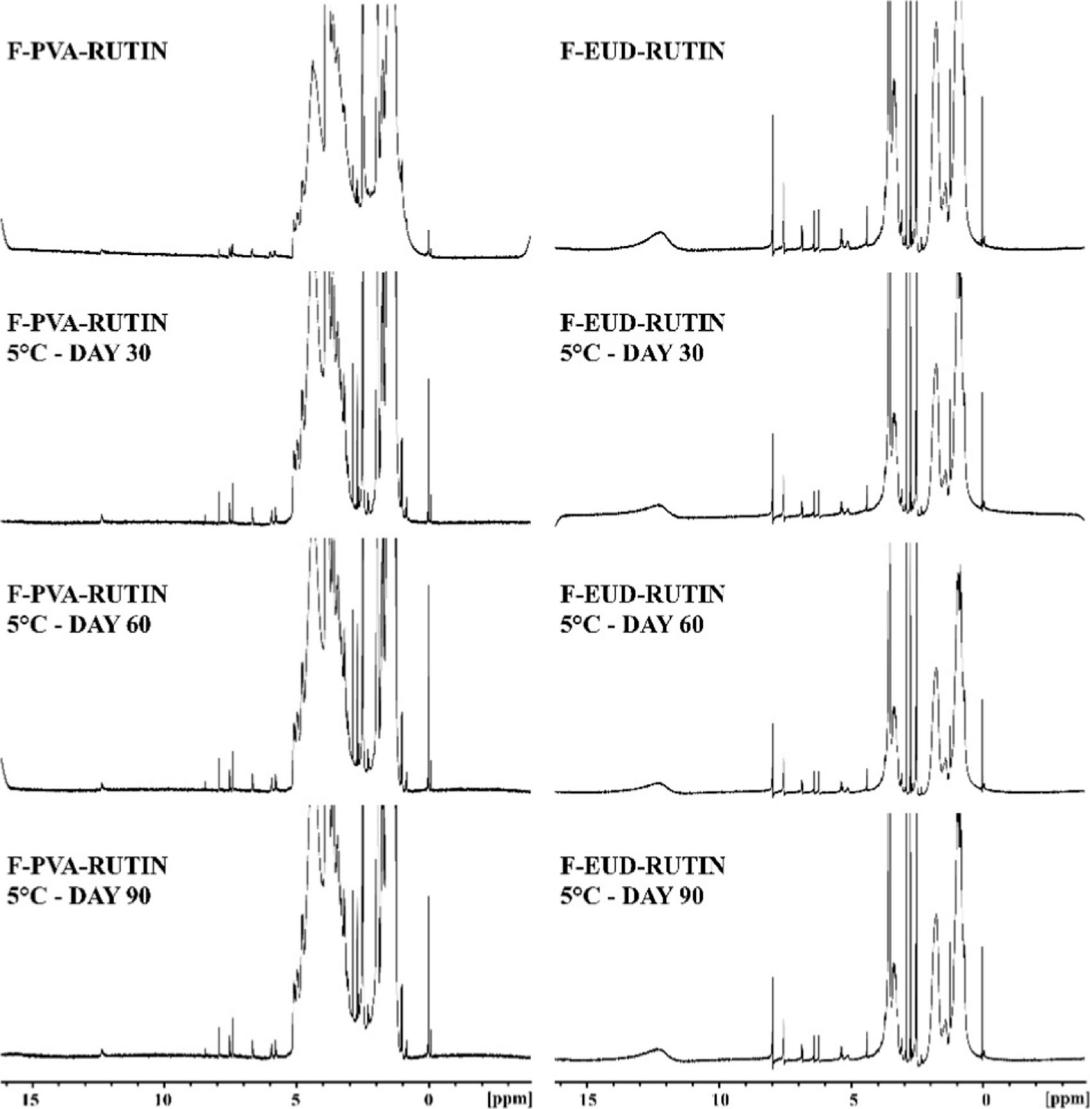
NMR spectra for stability studies (5 °C).

The animals were divided into the following treatment groups:

- Untreated control group.
- Positive control group, treated with Madecassol.
- Negative control groups, treated with blank formulations (F-PVA and F-EUD).
- Experimental groups, treated with active agent formulations (F-PVA-Rutin and F-EUD-Rutin).

The amount of Madecassol (∼25 mg) was adjusted to match the equivalent amount of active agent per unit wound area used in the active agent formulations.

Wound healing progress was monitored and photographed on days 0, 3, 7, 10, and 14, with wound areas calculated at each time point. On the specified days, four animals from each group were sacrificed, and wound tissue samples were collected for histological examination. Treatments were reapplied to the wounds on designated days to ensure consistency, with wound surfaces gently softened using a few drops of physiological saline prior to each application.

#### 3.5.1 Data Analysis

The software program GraphPad Prism ver. Nine was used for statistical analysis. The two-way Analysis of Variance (ANOVA) method and the Bonferroni technique were performed for the assessment of pharmacological effects.

## 4 Results and Discussion

### 4.1 Formulation Studies

Two polymers, PVA (Poly­(vinyl alcohol)) and Eudragit L100, were used in the formulation. PVA is ideal for electrospinning due to its moldability and surface tension reduction, functioning as a stabilizer.^23,24^ Its biocompatibility, biodegradability, bioadhesiveness, and moisture absorption make it suitable for wound dressings by effectively removing excess exudate and supporting tissue regeneration.^23^

Eudragit L100, a biocompatible, nonbiodegradable but pH-soluble hydrophobic polymer, was chosen for its mechanical strength, flexibility, and pH-sensitive nature, which accelerates drug release in high-pH environments.^18^ While healthy skin maintains an acidic pH of 5.5 to inhibit pathogens, injuries elevate the pH to 7.4 or higher, promoting pathogen growth.^25^ Eudragit L100s ability to respond to pH changes makes it ideal for wound dressings.^26^ Inflammation further increases wound pH, and Eudragit L100 facilitates enhanced drug release in response to this elevation, maximizing therapeutic efficacy.

Currently, no commercially available wound healing formulations contain Rutin hydrate, so its concentration in the electrospinning solution was determined based on data from various studies.

In a study producing Rutin-loaded chitosan oligosaccharide/polylactic acid (PCL) nanofibers, the active agent concentration in the electrospinning solution was calculated as 5% of the PCL content and 4.85% of the total polymer content.^27^ Similarly, a hydrogel study specified a Rutin concentration of 250 μg·g^–1^, with rats treated twice daily with 0.5 g of the formulation during in vivo wound healing experiments.^28^ Another hydrogel study set the Rutin concentration at 750 μg·mL^–1^, and the formulation was used to completely fill the wound area during in vivo tests.^29^ A Pickering emulsion study, on the other hand, specified a Rutin concentration of 250 μg·mL^–1^, with rats treated twice daily with 0.5 mL of the formulation in an excisional wound healing model.^30^

Based on these studies, the active agent concentration was set at 10% of the polymer content, which aligns with solubility data, ensuring effective dissolution of Rutin hydrate.

Taguchi methods, widely used across various fields, identify experimental noise to enhance product quality while minimizing variability, making them suitable for formulation development in this study.^27^

Four key electrospinning parameters—polymer concentration, solvent ratios, needle-collector distance, and needle diameter—were identified as quality factors. While the first two are well-studied, the latter were further investigated in this study due to limited literatüre.^15,31,32^

To overcome Rutin hydrate’s low water solubility, balance exudate, and support cell migration during reepithelialization, nanofibers with low diameter and high porosity were targeted, making fiber diameter a key quality parameter. Additionally, bead presence, which disrupts fiber uniformity and mechanical consistency, was analyzed as another critical quality factor.

The SEM images for the formulations prepared with PVA (P1–P16) and Eudragit L100 (E1–E16), which illustrate the morphological features and bead distribution of the electrospun nanofibers, are provided in Figures S2–S5 and S6–S9, respectively. These images illustrate the morphological characteristics of the electrospun nanofibers, including uniformity, surface texture, and bead formation. The corresponding average fiber diameters and bead counts obtained from these images are provided in Tables S3 and S4.

#### 4.1.1 Taguchi Analysis

The simultaneous optimization of minimizing fiber diameter and eliminating bead structures is essential because each factor significantly influences the functional performance of electrospun nanofibers. Achieving ultrafine fibers enhances the surface-to-volume ratio, improving their efficacy in applications such as drug delivery, tissue engineering, and filtration, by enabling better active-agent loading, controlled release, and improved cellular interactions. Conversely, bead formation represents structural defects, reducing mechanical stability, uniformity, and biocompatibility, thereby negatively impacting nanofiber functionality. Conducting both optimizations simultaneously, rather than separately, ensures accurate determination of optimal process parameters by considering potential interactions between variables, ultimately leading to structurally uniform, bead-free nanofibers with optimal diameters for advanced application performance.

Taguchi analysis revealed that the most significant and statistically effective parameter (*p* < 0.05) for fiber diameter and bead formation in nanofibers prepared with PVA was the PVA concentration. Detailed S/N ratio tables and ANOVA results are provided in Tables S5–S8. While lower PVA concentrations resulted in thinner fibers, concentrations of 950 mg or less led to bead formation, making 950 mg the minimum limit. Although bead-free fibers were successfully produced at PVA concentrations of 1000 mg or higher, additional experiments were conducted to determine the optimal concentration for producing the finest bead-free fibers. The PVA concentration was adjusted within the range of 950–1000 mg, while maintaining all other parameters at the levels suggested by the Taguchi analysis (Table 3). Representative SEM images from these trials are presented in Figure 2. Ultimately, the optimal PVA concentration was determined as 1000 mg, as it produced bead-free fibers.

**Table 3:** Parameters Examined for the Variation in Fiber Diameter Based on PVA Content

	PVA (mg)	DMF (mL)	BDW (mL)	Rutin hydrate (mg)	range (cm)	needle diameter (G)
PP1	987.5	6.5	6.5	98.75	13	23
PP2	975	6.5	6.5	97.5	13	23
PP3	962.5	6.5	6.5	96.25	13	23

For nanofibers prepared with Eudragit L100, fiber diameter was similarly influenced by polymer concentration (*p* < 0.05). However, unlike PVA-based formulations, bead formation in Eudragit L100-based nanofibers was statistically linked to both the polymer concentration and the DMF content in the solvent system (*p* < 0.05), as shown in Tables S9–S12. This correlation can be attributed to the higher surface tension of DMF at room temperature (0.9442 g·cm^–3^) compared to ethanol (0.7912 g·cm^–3^). Increasing the ethanol ratio in the solvent mix reduced surface tension, thereby minimizing bead formation.^33–35^

For Eudragit L100, lower concentrations produced thinner fibers, but at 1300 mg or less, bead formation occurred even at optimal DMF ratios. While bead-free fibers were observed at 1350 mg or higher, further tests were conducted with concentrations between 1300 mg and 1350 mg to obtain the thinnest bead-free fibers. Since DMF content had no statistically significant effect on fiber thickness (*p* > 0.05), the DMF ratio that minimized bead count and other parameters at levels yielding the smallest fiber diameters were fixed, and Eudragit L100 concentration was varied (Table 4, Figure 3). The optimal concentration of Eudragit L100 was determined as 1375 mg.

**Table 4:** Parameters Examined for the Variation in Fiber Diameter Based on Eudragit L100 Content

	EUD (mg)	MeOH (mL)	DMF (mL)	Rutin hydrate (mg)	range (cm)	needle diameter (G)
EE1	1387.5	7.5	2.5	138.75	11	23
EE2	1375	7.5	2.5	137.5	11	23
EE3	1362.5	7.5	2.5	136.25	11	23

As a result of the formulation studies, the electrospinning parameters for obtaining the thinnest bead-free fibers in optimal wound dressings were determined as shown in Tables 5 and 6. For the production of F-PVA and F-EUD nanofibers, which are formulations without active agent, the same parameters were applied without the addition of active agent.

**Table 5:** Optimum Electrospinning Parameters for Nanofibers Prepared with PVA

	PVA (mg)	DMF (mL)	BDW (mL)	Rutin hydrate (mg)	range (cm)	needle diameter (G)
F-PVA-Rutin	1000	6.5	6.5	100	13	23
F-PVA	1000	6.5	6.5	0	13	23

**Table 6:** Optimum Electrospinning Parameters for Nanofibers Prepared with Eudragit L100

	EUD (mg)	MeOH (mL)	DMF (mL)	Rutin hydrate (mg)	range (cm)	needle diameter (G)
F-EUD-Rutin	1375	7.5	2.5	137.5	11	23
F-EUD	1375	7.5	2.5	0	11	23

Electrospinning solutions containing PVA were spun at a flow rate of 3.5 mL/h and a voltage of 26 kV with a total volume of 6.5 mL, while solutions containing Eudragit L100 were spun at a flow rate of 3 mL/h and a voltage of 20 kV with a total volume of 3.64 mL.

The morphological properties of nanofibers play a critical role in determining their performance in wound healing applications. In this study, bead-free, uniform nanofibers were successfully fabricated for both PVA and Eudragit L100 formulations. The average diameters of 258.37 nm (F-PVA-Rutin) and 125.12 nm (F-EUD-Rutin) suggest a high surface area-to-volume ratio, which is beneficial for enhanced drug release, moisture regulation, and cellular interactions. The absence of bead formation is particularly important as beads can disrupt membrane uniformity, hinder drug diffusion, and compromise mechanical integrity. Therefore, the achieved nanofiber morphology is not only an indicator of optimized processing conditions but also a crucial factor that enhances the therapeutic potential and physical performance of the developed wound dressings.

### 4.2 Solubility of Rutin Hydrate

The solubility study revealed that the active agent exhibited solubilities of 0.113 ± 0.005 mg/mL in BDW, 0.118 ± 0.003 mg/mL in PBS, 42.750 ± 1.977 mg/mL in MeOH, and 37.387 ± 0.497 mg/mL in EtOH, as shown in Table 7. Additionally, when tested in DMF, the entire 0.4 g of the active agent dissolved completely, indicating that the solubility of Rutin hydrate in DMF is > 80 mg/mL. These results highlight the significantly higher solubility of the active agent in organic solvents such as MeOH and EtOH compared to aqueous solutions.

**Table 7:** Solubility Values of Rutin Hydrate in Different Solvents

		solubility according to days (mg·mL^–1^)			
days (*n* = 3)		BDW	PBS	MeOH	EtOH
first day	1	0.118	0.121	41.065	37,284
	2	0.119	0.126	40.494	37,674
	3	0.117	0.114	39.876	36,011
mean		0.118	0.120	40.478	36.990
SS		0.001	0.006	0.595	0.870
second day	1	0.114	0.117	46.642	38,981
	2	0.109	0.114	45.274	38,054
	3	0.110	0.113	40.332	36,791
mean		0.111	0.115	44.083	37.942
SS		0.003	0.002	3.319	1.099
third day	1	0.112	0.124	43.821	37,246
	2	0.102	0.114	46.488	37,954
	3	0.113	0.121	40.754	36,466
mean		0.109	0.120	43.688	37.222
SS		0.006	0.005	2.869	0.744
solubility		0.113 ± 0.005	0.118 ± 0.003	42.750 ± 1.977	37.387 ± 0.497

### 4.3 Characterization of Nanofibers

#### 4.3.1 Morphology

The F-PVA and F-EUD formulations exhibited an opaque white, smooth, and homogeneous appearance. The F-PVA-Rutin formulation displayed an opaque yellow appearance, while the F-EUD-Rutin formulation showed a yellowish, opaque white color due to Rutin hydrate dispersion in the Eudragit L100 matrix. The yellow color of the F-PVA-Rutin formulation, despite PVA’s transparency, is attributed to light diffraction within the nanofibers.

Morphological analysis using higher-resolution SEM, presented in Figures 4 and 5, revealed no bead formation in either formulation. The average fiber diameters were measured as 258.371 nm for F-PVA-Rutin and 125.115 nm for F-EUD-Rutin, with none of the fibers exceeding the micron scale.

#### 4.3.2 Spectroscopic Analysis

NMR analysis was performed to verify the chemical stability of the active compound (rutin hydrate) throughout the electrospinning process, ensuring no structural degradation occurred. FTIR analysis was conducted to confirm the successful encapsulation and physical integration of the active substance within the polymer matrices of the nanofibers.

FTIR spectra were used to assess the entrapment of the active ingredient within the polymers forming the nanofibers. As seen in Figure 6, the specific vibrations of Rutin hydrate were visible in the physical mixture spectra but absent in the FTIR spectra of the F-PVA-Rutin and F-EUD-Rutin formulations. This absence indicates that Rutin hydrate was successfully entrapped by the polymers in both formulations.^36^

During the electrospinning process, the stability of the active ingredient in the formulations was evaluated using comparative NMR results. As shown in Figure 7 the NMR spectra of the F-PVA-Rutin and F-EUD-Rutin formulations revealed the specific NMR peaks of the polymers alongside the characteristic peaks of Rutin hydrate. These results confirm that the formulation preparation steps did not alter the chemical structure of Rutin hydrate.

#### 4.3.3 Thermal Analysis

Another characteristic examined to evaluate the entrapment of the active ingredient within the polymers was the DSC thermograms. As shown in Figure 8, the melting peak of Rutin hydrate, which is present in the physical mixture thermograms, was absent in the DSC thermograms of the F-PVA-Rutin and F-EUD-Rutin formulations. This finding suggests that Rutin hydrate transitioned into an amorphous form in both formulations, further confirming its entrapment within the polymers.

#### 4.3.4 Texture Analysis

The mechanical properties of the F-PVA-Rutin and F-EUD-Rutin formulations were evaluated in terms of elongation at break and tensile strength. As shown in Scheme 1, the F-PVA-Rutin formulation exhibited higher elongation at break (7.156 ± 0.193 mm) and tensile strength (438.8 ± 17.33 g), indicating a more flexible and mechanically robust structure. In contrast, the F-EUD-Rutin formulation showed lower elongation at break (3.192 ± 0.111 mm) and tensile strength (155.46 ± 18.28 g), reflecting a comparatively more brittle structure.

**Scheme 1:**
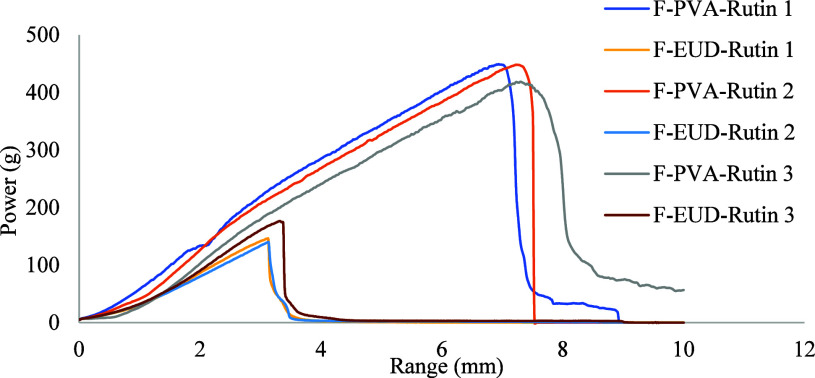
Mechanical Changes during the Texture Analysis of the Formulations (*n* = 3)

#### 4.3.5 Antioxidant Activity

The antioxidant activities of the formulations, as shown in Table 8, were tested using the DPPH method. Formulations with Rutin hydrate and F-PVA showed activity, while F-EUD showed none.

**Table 8:** Comparative Antioxidant Activity Test Results

formulation	scavenging %
Rutin hydrate	69.42 ± 0.6
F-PVA	5.68 ± 2.92
F-EUD	inactive
F-PVA-Rutin	73.61 ± 2.01
F-EUD-Rutin	70.23 ± 1.77

These findings are consistent with a previous study that evaluated the antioxidant properties of Rutin in its pure form and within nanofibers, reporting no significant changes in antioxidant activity.^37^ Notably, the DPPH radical scavenging activity of the F-PVA-Rutin formulation was higher compared to pure Rutin hydrate, likely due to the intrinsic antioxidant properties of PVA nanofibers, even in the absence of active ingredients.

#### 4.3.6 Drug Loading

The quantification of the active agent in the formulations showed that the F-PVA-Rutin formulation had an active agent content of 78.735 ± 2.307 μg/mg per mass and 87.983 ± 2.055 μg/cm^2^ per area. Similarly, the F-EUD-Rutin formulation contained 76.833 ± 2.238 μg/mg per mass and 85.807 ± 1.502 μg/cm^2^ per area.

The polymer was formulated with an initial drug-to-polymer ratio of 100 mg of active substance per gram of polymer, corresponding to an expected drug content of 100 mg within the total 1.1 g formulation. However, drug loading analysis revealed that the F-PVA-Rutin formulation contained 78.775 mg of active substance per gram of formulation, while the F-EUD-Rutin formulation exhibited a drug loading of 76.833 ± 2.238 mg/g. Based on these findings, the entrapment efficiency (EE) was calculated as 86.65% for the F-PVA-Rutin formulation and 84.52% for the F-EUD-Rutin formulation.

The observed ∼15% reduction in entrapment efficiency can be attributed to several factors. First, the standard deviation of approximately 3% in the drug quantification indicates potential variations in measurement precision. Additionally, trace amounts of residual solvent trapped within the nanofibers during electrospinning may contribute to discrepancies in drug loading. Furthermore, the transformation of the polymer from its bulk form to a nanofiber structure significantly increases the surface area-to-volume ratio, which enhances the moisture retention capacity of the nanofibers. Lastly, the adsorption of the drug onto, or its absorption by, compartments such as syringes and tubing used during the electrospinning process may further contribute to the observed drug loss.

#### 4.3.7 In Vitro Drug Release

The drug release study results (Scheme 2) showed extended release of the active agent from the formulations compared to pure Rutin hydrate. Mathematical modeling (Table 9) revealed Hixson–Crowell kinetics for pure Rutin hydrate, while F-PVA-Rutin and F-EUD-Rutin followed first-order kinetics. Although no clinical pharmacokinetic study was conducted, these findings suggest that nanofiber formulations enable sustained release aligned with first-order kinetics.

**Scheme 2:**
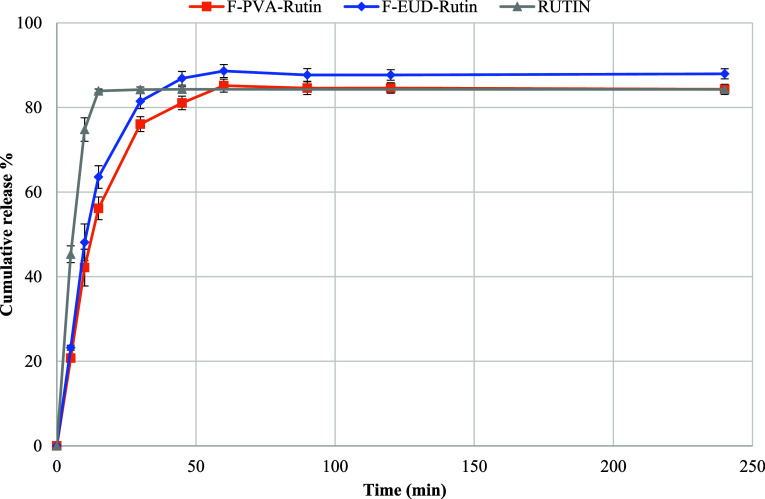
In Vitro Drug Release Scheme

**Table 9:** Mathematical Model Fitting of Release Profiles

		model		
formulation	evaluation	zero order	first order	Higuchi
F-PVA-Rutin	*r* ^2^	0.5919	0.9955	0.9513
	*k*	2.1330	0.0680	14.4500
	AIC	59.3255	27.8480	44.4464
F-EUD-Rutin	*r* ^2^	0.4987	0.9873	0.9276
	*k*	2.187	0.070	14.930
	AIC	60.9406	35.2343	47.3984
Rutin hydrate	*r* ^2^	0.8973	0.8923	0.9898
	*k*	7.571	0.306	26.217
	AIC	27.7152	27.9036	18.4870

		model		
formulation	evaluation	Hixson–Crowell	Baker–Lonsdale	Korsmeyer–Peppas
F-PVA-Rutin	*r* ^2^	0.9761	0.0000	0.8430
	*k*	0.016	0.009	10.826
	AIC	39.4610	84.0951	54.6402
F-EUD-Rutin	*r* ^2^	0.9825	0.0000	0.8437
	*k*	0.0226	0.011	16.723
	AIC	37.2096	85.1533	54.7810
Rutin hydrate	*r* ^2^	0.9968	0.0000	0.9894
	*k*	0.047	0.040	21.781
	AIC	13.8299	49.2411	20.6184

Drug release from nanofiber-based formulations often follows a first-order kinetic model, wherein the release rate is directly proportional to the remaining drug concentration within the nanofibers and gradually decreases over time. This behavior typically results in an initial burst release, followed by a sustained release phase. Such a profile is particularly advantageous in applications like wound healing or targeted drug delivery, where a rapid onset of action is desired initially, followed by prolonged therapeutic levels. However, as the drug content in the fibers diminishes, the release rate may decrease to subtherapeutic levels, potentially limiting efficacy. This phenomenon should be carefully considered during formulation design.^38^ The extended release profile could enhance therapeutic efficacy and reduce side effects.

### 4.4 Stability

The 3 month stability study, conducted under ICH-recommended conditions, evaluated formulations on the 30th, 60th, and 90th days. F-PVA and F-EUD retained their opaque white appearance, while F-PVA-Rutin and F-EUD-Rutin preserved their yellowish opaque look. FTIR, NMR, and DSC analyses showed no changes in peak structure or position, and active agent quantification along with fiber diameter measurements revealed no significant differences from the initial results (*p* > 0.05). These findings confirm the formulations’ stability over 90 days, ensuring consistency and reliability for product development and clinical use.

#### 4.4.1 Morphological Analysis

The formulations were microscopically analyzed only at the end of the 90th day, as shown in Figure 9. No statistically significant differences were observed in the fiber diameters between day 0 and day 90, as summarized in Table 10. These results confirm the morphological stability of the formulations over the study period.

**Table 10:** Fiber Diameter Results for Stability

	fiber diameter	
condition	F-PVA-Rutin	F-EUD-Rutin
day 0	258.37 ± 104.81	125.12 ± 63.37
5 °C	215.63 ± 73.96	136.92 ± 91.72
25 °C	295.4 ± 65.075	124.64 ± 70.84
40 °C	224.97 ± 53.41	144.79 ± 50.36

#### 4.4.2 Spectroscopic Analyses

The FTIR spectra of the formulations at 5 °C, 25 °C, and 40 °C are presented in Figures 10–12, respectively, while the corresponding NMR spectra are shown in Figures 13–15.

**14:**
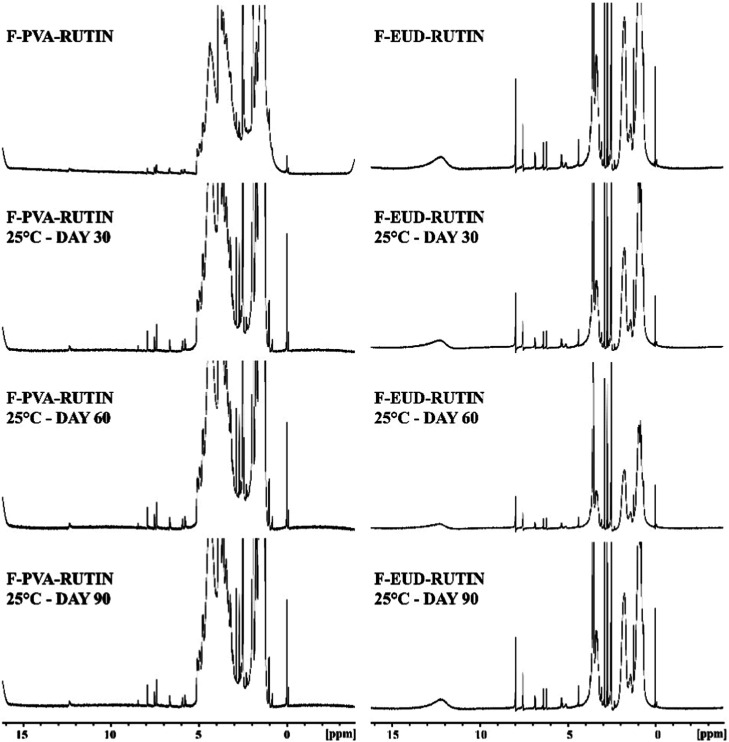
NMR spectra for stability studies (25 °C).

**Figure 15:**
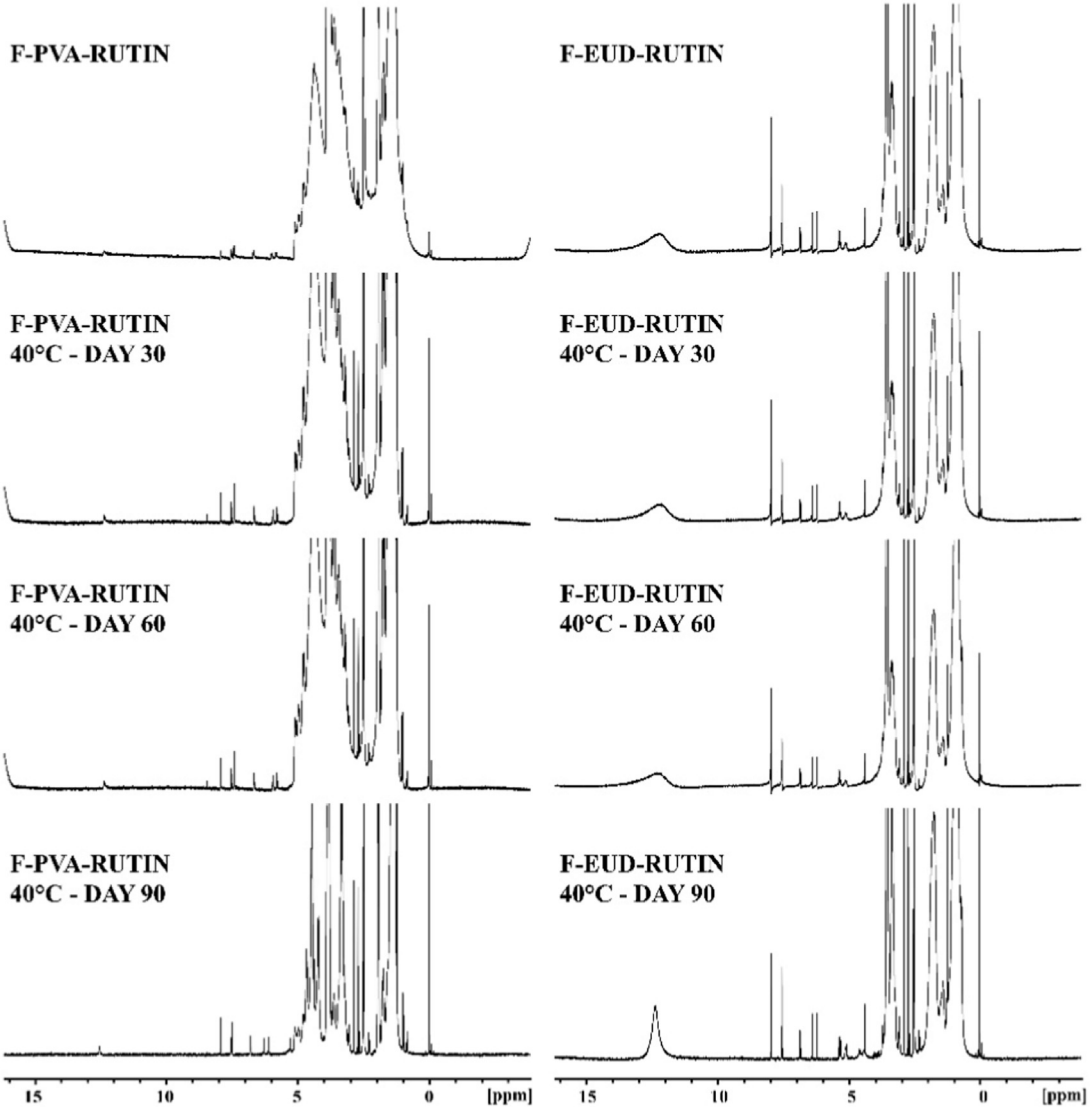
NMR spectra for stability studies (40 °C).

#### 4.4.3 Thermal Analysis

The comparative thermal analysis schemes of the formulations performed exclusively on the 90th day, as shown in Figure 16, indicate that no changes were observed in the amorphous structure of the formulations. This confirms that the formulations maintained their amorphous nature throughout the stability period.

**Figure 16:**
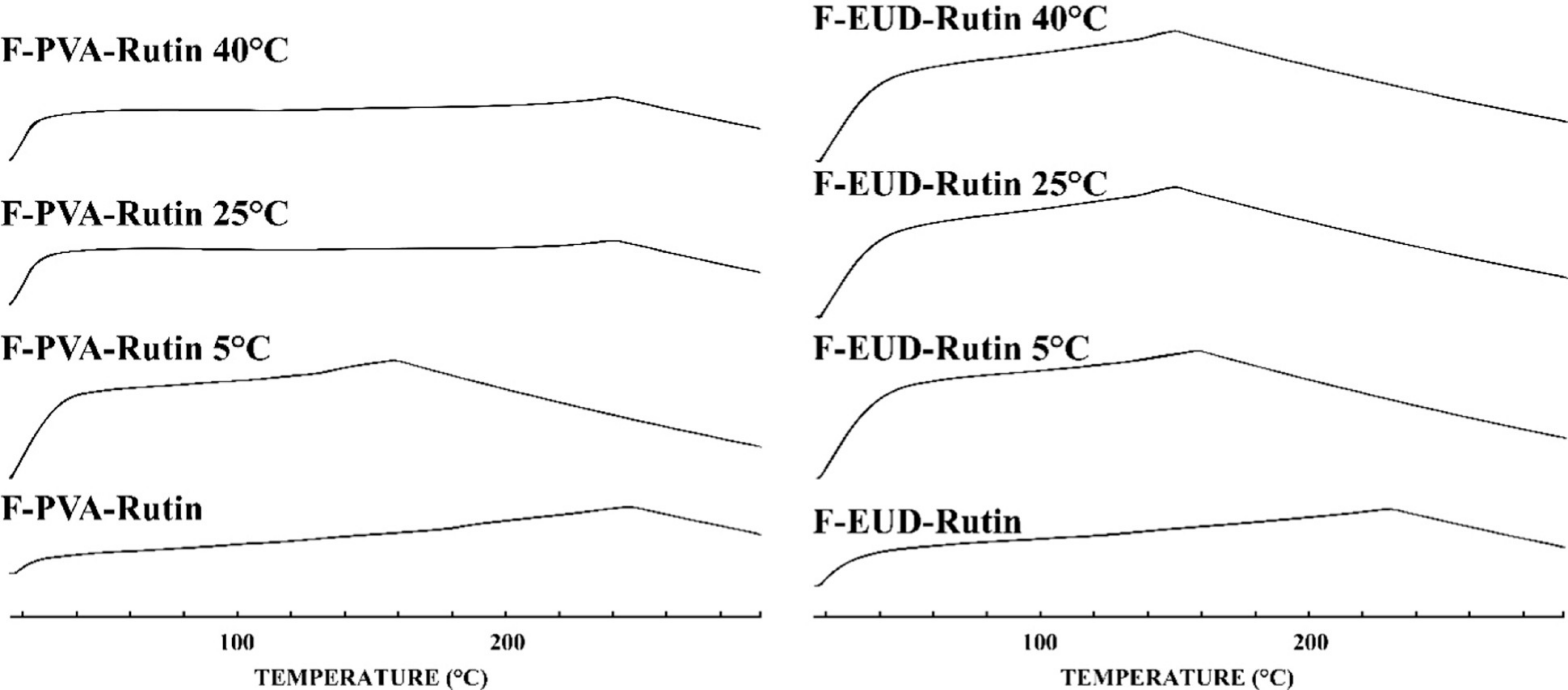
DSC thermograms for stability studies (40 °C).

#### 4.4.4 Antioxidant Activity

The results of the antioxidant activity test conducted exclusively on the 90th day, as presented in Table 11, indicate that the formulations retained their antioxidant activity throughout the stability period without any loss.

**Table 11:** Antioxidant Activity Results for Stability Study

formulation	scavenging %
F-PVA-Rutin	73.61 ± 2.01
F-PVA-Rutin 4 °C	73.97 ± 0.72
F-PVA-Rutin 25 °C	71.45 ± 2.5
F-PVA-Rutin 40 °C	71.72 ± 1
F-EUD-Rutin	70.23 ± 1.77
F-EUD-Rutin 4 °C	70.56 ± 0.42
F-EUD-Rutin 25 °C	71.79 ± 0.42
F-EUD-Rutin 40 °C	71.69 ± 2.17

#### 4.4.5 Drug Loading

The drug loading results of the F-PVA-Rutin and F-EUD-Rutin formulations remained within ±2% variation throughout the study period, indicating no statistically significant changes in the active agent content.

### 4.5 In Vivo Efficacy

The in vivo efficacy study was conducted using an excisional wound model, which is widely utilized for evaluating wound healing in vivo settings. This model enables the assessment of wound closure over time (Figure 17 and Scheme 3) and provides insights into critical processes such as granulation tissue formation, restoration of the vascular network, re-epithelialization by keratinocytes, and collagen deposition by fibroblasts and myofibroblasts (Figures 18 and 19).^39^

**Figure 17:**
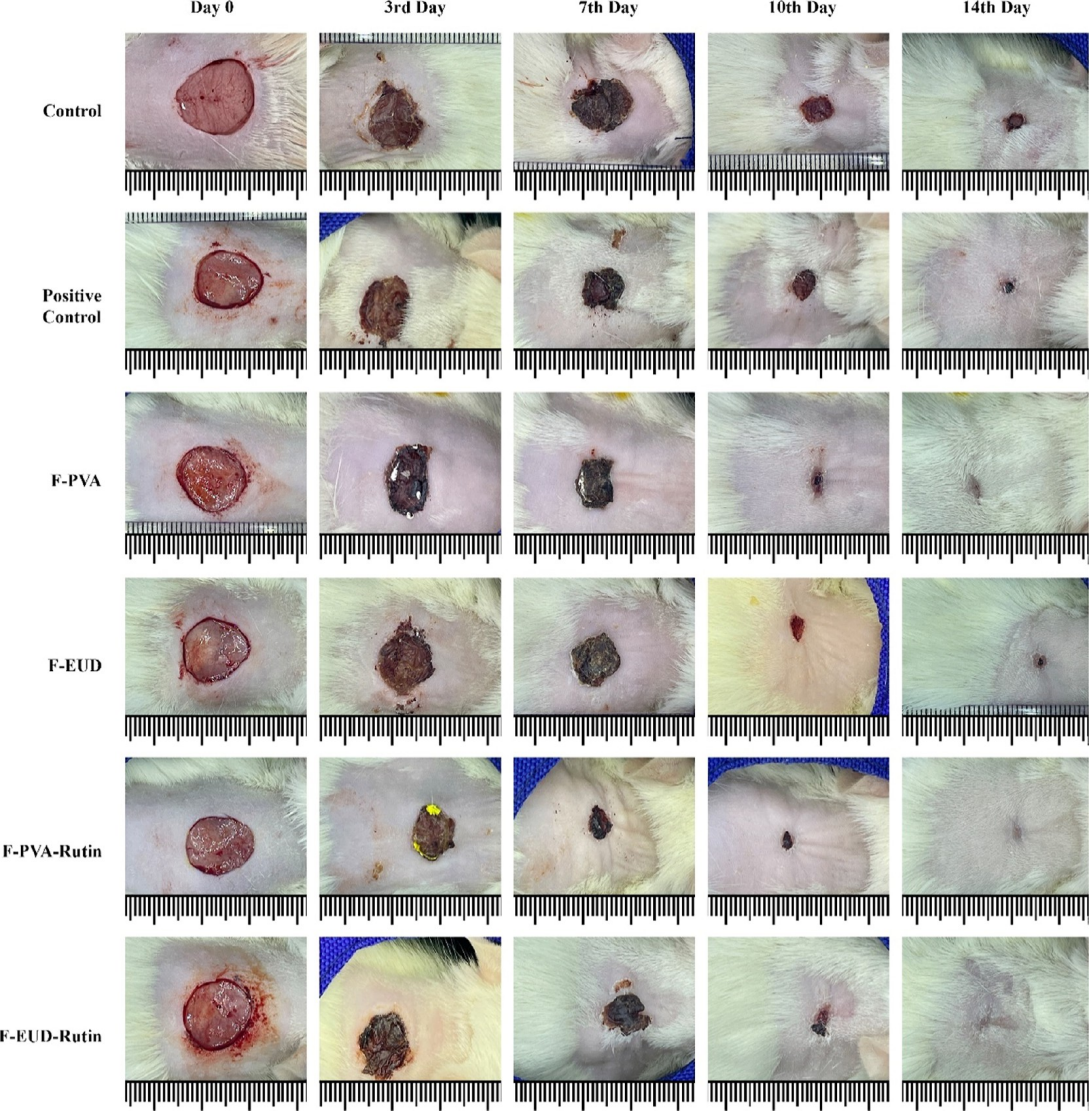
Representative images of the in vivo efficacy study (photographs courtesy of Dr. Sinan Özer. Copyright 2025).

**Scheme 3:**
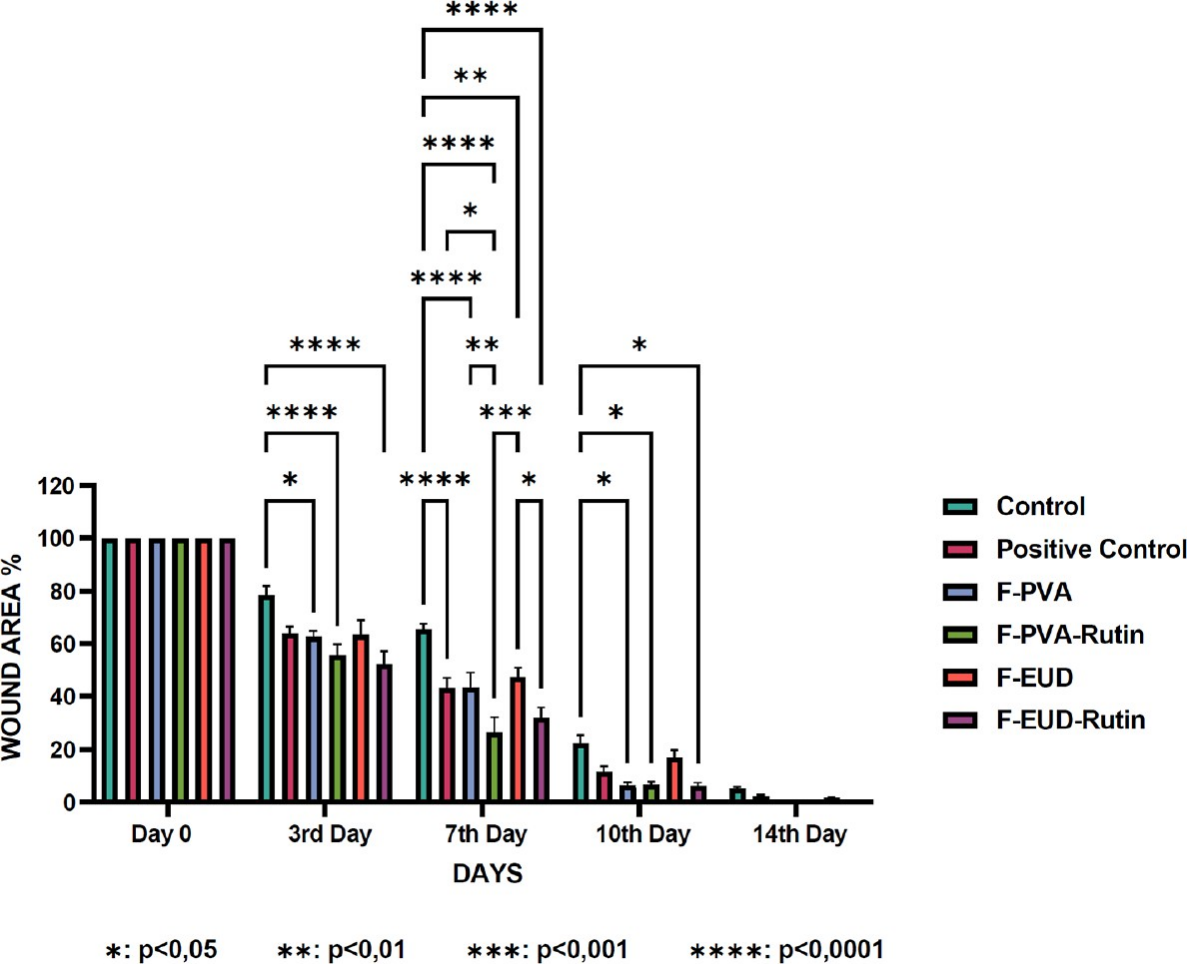
ANOVA Statistical Results Showing Percentage Reduction in Wound Area over Time for the In Vivo Efficacy Study

**Figure 18:**
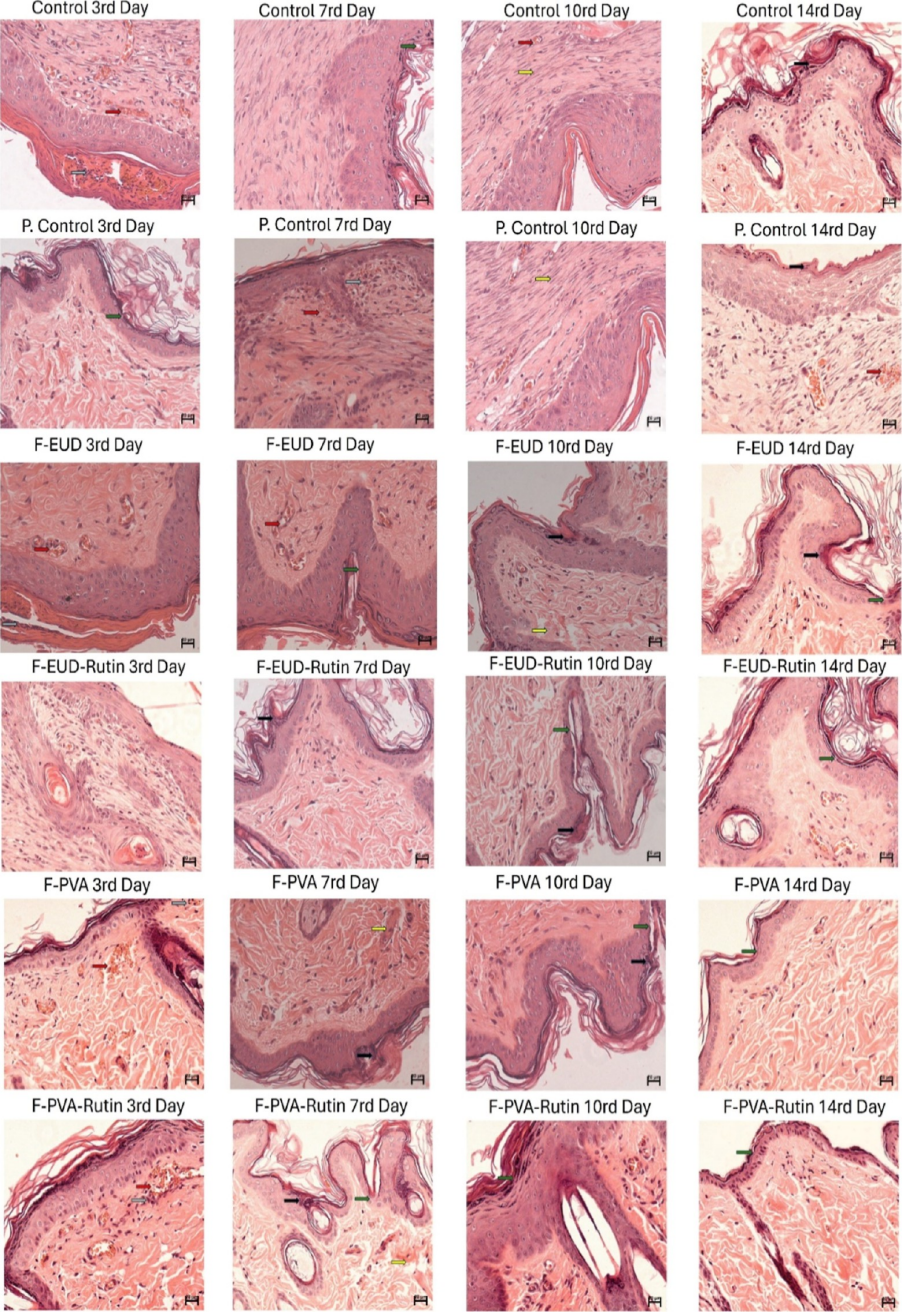
Histological evaluation of tissue samples collected on days 3, 7, 10, and 14 using hematoxylin & eosin staining to assess cellular morphology, tissue regeneration, and inflammatory response in wound healing (green arrow: re-epithelialization, yellow arrow: collagen tissue disorganization, gray arrow: neutrophil infiltration, red arrow: hemorrhage, black arrow: granulation tissue formation).

**Figure 19:**
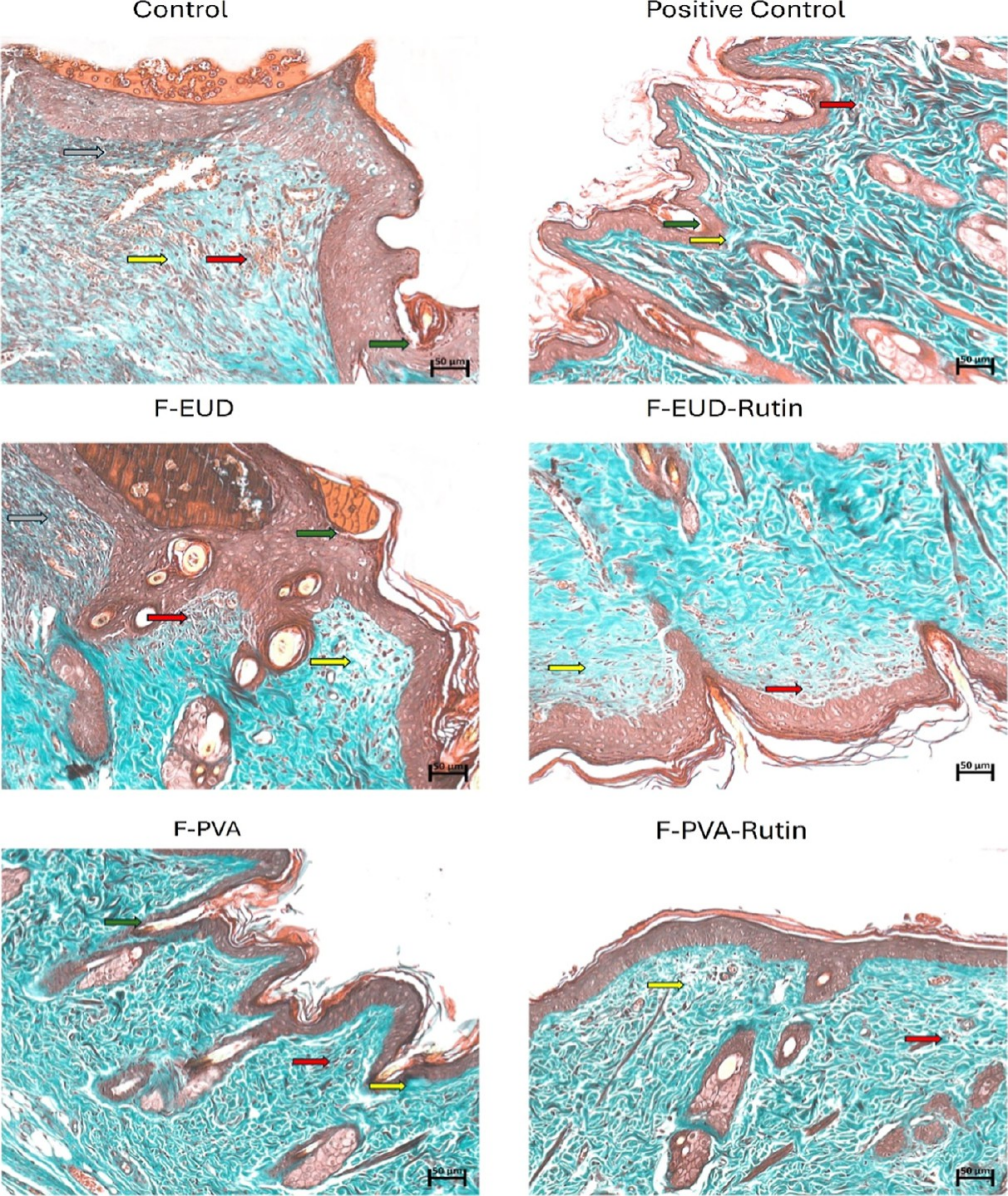
Histological evaluation of tissue samples collected on day 14 using Masson’s trichrome staining to assess collagen deposition, ECM remodeling, and overall tissue regeneration in wound healing. (Green arrow: re-epithelialization; yellow arrow: collagen tissue disorganization; gray arrow: neutrophil infiltration; red arrow: hemorrhage; black arrow: granulation tissue formation.)

On day 3, significant reductions in wound area reduction were observed for the F-PVA formulation (*p* < 0.05) and the active agent-containing formulations, F-PVA-Rutin and F-EUD-Rutin (*p* < 0.0001). The superior performance of the active agent formulations during the inflammatory phase of wound healing is attributed to the anti-inflammatory properties of Rutin hydrate.^40^ Histological analysis further supported this observation, with significant reductions in neutrophil infiltration, particularly for the F-EUD-Rutin nanofiber formulation, on day 3.

By day 7, all groups exhibited significant wound area reduction compared to the control group. The active agent-containing formulations demonstrated greater efficacy than the placebo formulations (*p* < 0.0001 for positive control, F-PVA, F-PVA-Rutin, and F-EUD-Rutin; *p* < 0.01 for F-EUD). Furthermore, F-PVA-Rutin showed significant improvement over F-PVA (*p* < 0.01) and the positive control (*p* < 0.05), while F-EUD-Rutin outperformed F-EUD (*p* < 0.05). This phase of healing, characterized by ongoing neutrophil infiltration, granulation tissue formation, and neovascularization, highlighted the role of Rutin hydrate in supporting granulation tissue development and angiogenesis.

Although no statistically significant differences in granulation tissue formation were observed at a 95% confidence interval due to high standard deviations, the averages of both active agent and placebo formulations were higher than the control and positive control groups. The anti-inflammatory properties of Rutin hydrate were noted to contribute to granulation tissue formation. Furthermore, the nanofiber structure likely provided an ECM-like scaffold that enhanced fibroblast activity, thereby supporting the development of granulation tissue.

Regarding angiogenesis, despite Rutin’s known inhibitory effect on VEGF expression,^7^ F-PVA-Rutin and F-EUD-Rutin demonstrated statistically significant improvements in neovascularization. This effect is likely due to the positive impact of Rutin hydrate and the nanofiber structure on granulation tissue formation and collagen synthesis, creating scaffolds that facilitate vascular endothelial cell migration.

On day 10, the active agent formulations and the F-PVA formulation showed statistically significant wound area reduction compared to the control group (*p* < 0.05). In terms of collagen tissue development, no significant differences were observed between the control and positive control groups, but the active agent formulations exhibited significant improvements. Rutin hydrate’s well-documented role in promoting collagen synthesis^41^ was evident in these results.

By day 14, statistical significance in wound healing was no longer observed as the wounds had nearly completely healed. Nevertheless, visual assessments indicated better outcomes in all experimental groups compared to the control. Histological evaluations on day 14 revealed similar findings to those observed on day 10. The F-PVA-Rutin showing statistically significant effects on angiogenesis, while the placebo groups had no notable impact. Although the positive control and F-EUD-Rutin groups did not exhibit statistical significance, their average performance was superior to other groups.

Overall, the study demonstrated that Rutin hydrate positively influenced various stages of wound healing, particularly through its anti-inflammatory properties, enhancing neutrophil infiltration, granulation tissue formation, and collagen synthesis. The nanofiber-based delivery systems further supported granulation tissue development and angiogenesis due to their scaffold-like structure.

Manjit et al. (2024) evaluated the fabrication of dual drug-loaded polycaprolactone–gelatin composite nanofibers for diabetic wound healing.^42^ Their results demonstrated 83.3% wound closure by day 14, surpassing the marketed formulation (CIPLADINE, 73.9%). By day 21, nanofiber-treated wounds reached 97.5% healing, highlighting their superior efficacy in diabetic wound management, particularly in preventing fluid retention and sepsis.

In comparison, our study focuses on rutin hydrate-loaded nanofibers (F-PVA-Rutin, F-EUD-Rutin), which significantly accelerated wound contraction, granulation, and angiogenesis. By day 7 and 10, rutin-loaded formulations showed enhanced healing over controls (*p* < 0.0001), attributed to rutin’s anti-inflammatory and collagen-promoting effects. Specifically, wound areas in the F-PVA-Rutin group were approximately 39% smaller than the control on day 7 and 16% smaller on day 10, while the F-EUD-Rutin group exhibited reductions of 33.5% and 16% on the same days, respectively. Although wound closure was nearly complete by day 14, our findings emphasize histological improvements and tissue remodeling rather than late-stage healing percentages.

Both studies reinforce nanofiber-based scaffolds as advanced wound healing strategies, yet differ in their therapeutic approach. While Manjit et al. focus on dual-drug efficacy and clinical wound parameters, our study provides deeper histological insights into inflammation resolution and granulation tissue formation. Future research should explore long-term tissue regeneration and comparative efficacy in chronic wound models.

## 5 Conclusion

This study successfully developed and evaluated two innovative nanofiber-based wound dressing formulations containing rutin hydrate. The nanofiber systems, irrespective of the active ingredient and polymer type, exhibited remarkable wound healing properties, attributed to their unique structural and functional features. Nanofiber-based wound dressings incorporating rutin could offer a novel approach to wound care, addressing limitations in current topical treatments that may lead to allergic skin reactions, and providing a significant benefit to modern medicine. The optimization of electrospinning parameters using the Taguchi method ensured the production of nanofibers with reduced fiber diameters and a uniform, bead-free morphology, highlighting the precision and reliability of the process.

Comprehensive characterization analyses confirmed the successful integration of the active ingredient into the polymer matrix while preserving its chemical integrity. Stability assessments demonstrated that the formulations maintained their structural and functional properties over time, ensuring long-term reliability and effectiveness.

In vivo efficacy studies further emphasized the superior wound healing performance of the optimized formulations compared to both control and positive control groups. The formulations facilitated improved tissue regeneration and sustained release of the active ingredient, demonstrating their potential to significantly enhance wound healing outcomes.

To advance this research, future investigations should focus on developing sterilization protocols, exploring the compatibility of this delivery system with other active compounds, and integrating alternative polymers for extended-release profiles. These findings represent a significant step forward in the development of advanced wound care solutions, combining innovative biomaterial technologies with practical therapeutic applications to address persistent challenges in chronic wound management.

## Supplementary Material



## Notes

Extended experimental protocols, Taguchi optimization tables, SEM images of all fiber formulations (PDF)

## References

[1] Gonzalez A. C. d. O., Costa T. F., Andrade Z. d. A., Medrado A. R. A. P. (2016). Wound healing-A
literature review. An. Bras. Dermatol..

[2] Franz M. G. (2008). Guidelines
to aid healing of acute wounds by decreasing impediments of healing. Wound Repair Regen..

[3] Gurtner G. C., Werner S., Barrandon Y., Longaker M. T. (2008). Wound repair and
regeneration. Nature.

[4] Sen C. K. (2021). Human wound
and its burden: updated 2020 compendium of estimates. Adv. Wound Care.

[5] Global Wound Care Market Size By Product Type (Dressings, Advanced Wound Care Products), By Wound Type (Acute Wounds, Chronic Wounds), By End User (Hospitals, Home Healthcare), By Distribution Channel (Direct Sales, Retail Pharmacies), By Material Type (Natural Polymers, Synthetic Polymers), By Geographic Scope And Forecast, 2023 https://www.verifiedmarketreports.com/product/global-wound-care-market-size-and-forecast-to-2025/.

[6] Ganeshpurkar A., Saluja A. K. (2017). The pharmacological
potential of rutin. Saudi Pharm. J..

[7] Chen L.-Y., Huang C. N., Liao C. K., Chang H. M., Kuan Y. H., Tseng T. J., Yen K. J., Yang K. L., Lin H. C. (2020). Effects
of rutin on wound healing in hyperglycemic rats. Antioxidants.

[8] Gullon B., Lú-Chau T. A., Moreira M. T., Lema J. M., Eibes G. (2017). Rutin: A review
on extraction, identification and purification methods, biological
activities and approaches to enhance its bioavailability. Trends Food Sci. Technol..

[9] Bombin A. D. J., Dunne N. J., McCarthy H. O. (2020). Electrospinning
of natural polymers
for the production of nanofibres for wound healing applications. Mater. Sci. Eng., C.

[10] Dhondale M. R., Manjit M., Jha A., Kumar M., Bharti K., Kumar D., Mishra B. (2024). Heparin sodium
enriched gelatin/polycaprolactone
based multi-layer nanofibrous scaffold for accelerated wound healing
in diabetes. RSC Pharm..

[11] Ambekar R. S., Kandasubramanian B. (2019). Advancements in nanofibers for wound dressing: A review. Eur. Polym. J..

[12] Chouhan D., Mandal B. B. (2020). Silk biomaterials
in wound healing and skin regeneration
therapeutics: From bench to bedside. Acta Biomater..

[13] Zhang X., Wang Y., Gao Z., Mao X., Cheng J., Huang L., Tang J. (2024). Advances in wound dressing
based
on electrospinning nanofibers. J. Appl. Polym.
Sci..

[14] John J. V., McCarthy A., Karan A., Xie J. (2022). Electrospun nanofibers
for wound management. ChemNanoMat.

[15] Horuz T. İ., Belibağlı K. B. (2017). Production of electrospun
gelatin
nanofibers: an optimization study by using Taguchi’s methodology. Mater. Res. Express.

[16] Khanlou H. M., Ang B. C., Talebian S., Afifi A. M., Andriyana A. (2015). Electrospinning
of polymethyl methacrylate nanofibers: optimization of processing
parameters using the Taguchi design of experiments. Text. Res. J..

[17] Huang C.-Y., Hu K.-H., Wei Z.-H. (2016). Comparison
of cell behavior on pva/pva-gelatin
electrospun nanofibers with random and aligned configuration. Sci. Rep..

[18] Ao F. vd., Shen W., Ge X., Wang L., Ning Y., Ren H., Fan G., Huang M. (2020). Effects of the crystallinity on quercetin
loaded the Eudragit L-100 electrospun nanofibers. Colloids Surf., B.

[19] Yenilmez E., Yazan Y. (2017). Formulation, Chracterization
and In Vivo Efficacy of α-Tocopherol
Imprinted Polymeric System for Cosmetic Application. Eur. Int. J. Sci. Technol..

[20] ICH Guideline . Validation Of Analytical Procedures: Text And Methodology. Q2(R1), 2005 http://www.ich.org/fileadmin/Public_Web_Site/ICH_Products/Guidelines/Quality/Q2_R1/Step4/Q2_R1__Guideline.pdf.

[21] ICH Guideline . Stability Testing of New Drug Substances and Products. Q1A (R2), Current Step c, 2003 https://database.ich.org/sites/default/files/Q1BGuideline.pdf.

[22] Sami D. G., Heiba H. H., Abdellatif A. (2019). Wound healing
models: A systematic
review of animal and non-animal models. Wound
Med..

[23] Jalalian M., Mirkazemi S. M., Alamolhoda S. (2016). The effect of poly vinyl alcohol
(PVA) surfactant on phase formation and magnetic properties of hydrothermally
synthesized CoFe2O4 nanoparticles. J. Magn.
Magn. Mater..

[24] Scholes P. D., Coombes A., Illum L., Davis S., Watts J., Ustariz C., Vert M., Davies M. (1999). Detection and determination
of surface levels of poloxamer and PVA surfactant on biodegradable
nanospheres using SSIMS and XPS. J. Controlled
Release.

[25] Schneider L. A., Korber A., Grabbe S., Dissemond J. (2007). Influence
of pH on wound-healing: a new perspective for wound-therapy?. Arch. Dermatol. Res..

[26] Lee S. H., Bajracharya R., Min J. Y., Han J. W., Park B. J., Han H. K. (2020). Strategic
approaches for colon targeted drug delivery:
an overview of recent advancements. Pharmaceutics.

[27] Karna S. K., Sahai R. (2012). An overview on Taguchi method. Int. J. Eng.
Math. Sci..

[28] Almeida J. S. vd., Benvegnú D. M., Boufleur N., Reckziegel P., Barcelos R. C. S., Coradini K., de Carvalho L. M., Bürger M. E., Beck R. C. R. (2012). Hydrogels containing
rutin intended
for cutaneous administration: efficacy in wound healing in rats. Drug Dev. Ind. Pharm..

[29] Tran N. Q., Joung Y. K., Lih E., Park K. D. (2011). In situ forming
and rutin-releasing chitosan hydrogels as injectable dressings for
dermal wound healing. Biomacromolecules.

[30] Asfour M. H., Elmotasem H., Mostafa D. M., Salama A. A. A. (2017). Chitosan based
Pickering emulsion as a promising approach for topical application
of rutin in a solubilized form intended for wound healing: in vitro
and in vivo study. Int. J. Pharm..

[31] Abbas J. A. (2018). Electrospinning
of polyethylene terephthalate (PET) nanofibers: Optimization study
using taguchi design of experiment. IOP Conf.
Ser.: Mater. Sci. Eng..

[32] Celep G. K., Dincer K. (2017). Optimization of parameters
for electrospinning of polyacrylonitrile
nanofibers by the Taguchi method. Int. Polym.
Process..

[33] Gonçalves F. A.
M. M., Trindade A. R., Costa C., Bernardo J., Johnson I., Fonseca I., Ferreira A. (2010). PVT, viscosity, and surface tension
of ethanol: New measurements and literature data evaluation. J. Chem. Thermodyn..

[34] Haider A., Haider S., Kang I.-K. (2018). A comprehensive review summarizing
the effect of electrospinning parameters and potential applications
of nanofibers in biomedical and biotechnology. Arabian J. Chem..

[35] Ibrahim H. M., Klingner A. (2020). A review on electrospun
polymeric nanofibers: Production
parameters and potential applications. Polym.
Test..

[36] Yurtdaş
Kirimlioğlu G., Özer S., Büyükköroğlu G., Yazan Y. (2018). Formulation and in vitro evaluation of moxifloxacin hydrochloride-loaded
polymeric nanoparticles for ocular application. Lat. Am. J. Pharm..

[37] Zhou L. vd., Cai L., Ruan H., Zhang L., Wang J., Jiang H., Wu Y., Feng S., Chen J. (2021). Electrospun chitosan oligosaccharide/polycaprolactone
nanofibers loaded with wound-healing compounds of Rutin and Quercetin
as antibacterial dressings. Int. J. Biol. Macromol..

[38] Paarakh M. P., Jose P. A., Setty C. M., Peterchristoper G. V. (2018). Release
kinetics–concepts and applications. Int.
J. Pharm. Res. Technol..

[39] Baktır G. (2020). Wound repair
and experimental wound models. Experimed.

[40] Yoo H., Ku S.-K., Baek Y.-D., Bae J.-S. (2014). Anti-inflammatory
effects of rutin on HMGB1-induced inflammatory responses in vitro
and in vivo. Inflamm. Res..

[41] Anagnos D. (2018). Influence of biofield treated vitamin D3 on proliferation,
differentiation,
and maturation of bone-related parameters in MG-63 cell-line. Int. J. Biomed. Eng. Clin. Sci..

[42] Manjit M. vd., Kumar M., Kumar K., Dhondale M. R., Jha A., Bharti K., Rain Z., Prakash P., Mishra B. (2024). Fabrication
of dual drug-loaded polycaprolactone–gelatin composite nanofibers
for full thickness diabetic wound healing. Ther.
Delivery.

