# Supplementary material for: Development of Nanofiber Patch Formulation Containing Rutin Hydrate, In Vitro and In Vivo Evaluation
Source: ACS Omega. 2025 Jun 30;10(27):29037–58. doi: 10.1021/acsomega.5c01101 (PMC12268395; doi:10.1021/acsomega.5c01101)
Supplement: Supplementary file 1 [file ao5c01101_si_001.pdf]

# DEVELOPMENT OF NANOFIBER PATCH FORMULATION CONTAINING RUTIN HYDRATE, *IN VITRO* AND *IN VIVO* EVALUATION

Sinan Özer*[1], Evrim Yenilmez[1], Rana Arslan[2], Neziha Sinem Arı[3]

[1]*Anadolu University, Faculty of Pharmacy, Department of Pharmaceutical Technology*

[2]*Anadolu University, Faculty of Pharmacy, Department of Pharmacology*

[3]*Kütahya Health Sciences University, Faculty of Medicine, Department of Basic Medical Sciences*

## Abstract

Wound healing is a dynamic and multifactorial process that can be significantly impaired by oxidative stress, microbial infection, and chronic systemic conditions, often resulting in delayed recovery and poor tissue regeneration. This study investigates the use of rutin hydrate, a bioactive flavonoid with antioxidant and collagen-promoting effects, in electrospun nanofiber dressings to enhance wound repair outcomes.

Electrospinning technology was used to fabricate nanofibers from polyvinyl alcohol (PVA) and Eudragit L100, with optimized parameters determined via the Taguchi method. The nanofibers had average diameters of 258.371 nm (PVA) and 125.115 nm (Eudragit), with drug loading capacities of 78.735 ± 2.307 µg/mg per mass and 87.983 ± 2.055 µg/cm² per area (PVA); 76.833 ± 2.238 µg/mg per mass and 85.807 ± 1.502 µg/cm² per area (Eudragit). Characterization via SEM, FTIR, DSC, and NMR confirmed uniform, bead-free nanofibers with enhanced stability and controlled drug release. In vitro studies showed first-order drug release kinetics (Hixson-Crowell model), balancing burst release with sustained delivery.

In vivo wound healing in rats demonstrated significantly faster recovery with rutin-loaded nanofibers ($p < 0.0001$ for F-PVA-Rutin and F-EUD-Rutin on day 3 and day 7). Histological analysis revealed reduced neutrophil infiltration, enhanced granulation tissue, and improved angiogenesis, confirming the therapeutic efficacy of rutin.

These findings support the potential of rutin-loaded electrospun nanofiber dressings as an effective and scalable approach for promoting wound healing through localized, sustained drug delivery.

**Table S1.** Component Quantities for Formulations Using PVA

| | PVA (mg) | DMF (mL) | BDW (mL) | Rutin hydrate (mg) | Range (cm) | Needle Diameter (G) |
|---|---|---|---|---|---|---|
| **P1** | 1050 | 5 | 8 | 105 | 17 | 17 |
| **P2** | 1050 | 5.5 | 7.5 | 105 | 15 | 19 |
| **P3** | 1050 | 6 | 7 | 105 | 13 | 21 |
| **P4** | 1050 | 6.5 | 6.5 | 105 | 11 | 23 |
| **P5** | 1000 | 5 | 8 | 100 | 15 | 21 |
| **P6** | 1000 | 5.5 | 7.5 | 100 | 17 | 23 |
| **P7** | 1000 | 6 | 7 | 100 | 11 | 17 |
| **P8** | 1000 | 6.5 | 6.5 | 100 | 13 | 19 |
| **P9** | 950 | 5 | 8 | 95 | 13 | 23 |
| **P10** | 950 | 5.5 | 7.5 | 95 | 11 | 21 |
| **P11** | 950 | 6 | 7 | 95 | 17 | 19 |
| **P12** | 950 | 6.5 | 6.5 | 95 | 15 | 17 |
| **P13** | 900 | 5 | 8 | 90 | 11 | 19 |
| **P14** | 900 | 5.5 | 7.5 | 90 | 13 | 17 |
| **P15** | 900 | 6 | 7 | 90 | 15 | 23 |
| **P16** | 900 | 6.5 | 6.5 | 90 | 17 | 21 |

**Table S2**. Component Quantities for Formulations Using Eudragit L100

| | EUD (mg) | MeOH (mL) | DMF (mL) | Rutin hydrate (mg) | Range (cm) | Needle Diameter (G) |
|---|---|---|---|---|---|---|
| **E1** | 1400 | 6 | 4 | 140 | 11 | 17 |
| **E2** | 1400 | 6.5 | 3.5 | 140 | 13 | 19 |
| **E3** | 1400 | 7 | 3 | 140 | 15 | 21 |
| **E4** | 1400 | 7.5 | 2.5 | 140 | 17 | 23 |
| **E5** | 1350 | 6 | 4 | 135 | 13 | 21 |
| **E6** | 1350 | 6.5 | 3.5 | 135 | 11 | 23 |
| **E7** | 1350 | 7 | 3 | 135 | 17 | 17 |
| **E8** | 1350 | 7.5 | 2.5 | 135 | 15 | 19 |
| **E9** | 1300 | 6 | 4 | 130 | 15 | 23 |
| **E10** | 1300 | 6.5 | 3.5 | 130 | 17 | 21 |
| **E11** | 1300 | 7 | 3 | 130 | 11 | 19 |
| **E12** | 1300 | 7.5 | 2.5 | 130 | 13 | 17 |
| **E13** | 1250 | 6 | 4 | 125 | 17 | 19 |
| **E14** | 1250 | 6.5 | 3.5 | 125 | 15 | 17 |
| **E15** | 1250 | 7 | 3 | 125 | 13 | 23 |
| **E16** | 1250 | 7.5 | 2.5 | 125 | 11 | 21 |

**Table S3.** Fiber diameters and bead counts for nanofibers prepared with PVA polymer

| Form. | Avg. Fiber Diameter (nm) | Bead Count |
|---|---|---|
| **P1** | 276.933 | 0 |
| **P2** | 363.800 | 0 |
| **P3** | 260.400 | 0 |
| **P4** | 244.533 | 0 |
| **P5** | 254.733 | 0 |
| **P6** | 231.733 | 0 |
| **P7** | 301.333 | 0 |
| **P8** | 216.933 | 0 |
| **P9** | 151.067 | 62 |
| **P10** | 171.427 | 72 |
| **P11** | 183.040 | 60 |
| **P12** | 203.200 | 34 |
| **P13** | 181.467 | 55 |
| **P14** | 137.486 | 152 |
| **P15** | 145.520 | 105 |
| **P16** | 166.000 | 149 |

**Table S4.** Fiber diameters and bead counts for nanofibers prepared with Eudragit L100 polymer

| Form. | Avg. Fiber Diameter (nm) | Bead Count |
|---|---|---|
| **P1** | 147.600 | 7 |
| **P2** | 153.493 | 0 |
| **P3** | 166.773 | 2 |
| **P4** | 175.400 | 0 |
| **P5** | 133.300 | 22 |
| **P6** | 125.680 | 19 |
| **P7** | 129.173 | 9 |
| **P8** | 144.560 | 0 |
| **P9** | 103.207 | 38 |
| **P10** | 119.433 | 28 |
| **P11** | 119.493 | 11 |
| **P12** | 125.727 | 7 |
| **P13** | 103.600 | 57 |
| **P14** | 101.420 | 28 |
| **P15** | 101.640 | 30 |
| **P16** | 90.607 | 21 |

**Table S5.** Response Table for S/N Ratios of Nanofiber Diameter Taguchi Analysis (Prepared with PVA)

| Level | PVA Amount (mg) | Water Volume in Solvent (mL) | Range (cm) | Needle Diameter (G) |
|---|---|---|---|---|
| 1 | -43.9 | -46.26 | -46.8 | -46.84 |
| 2 | -44.92 | -46.6 | -45.35 | -47.09 |
| 3 | -47.93 | -46.49 | -47.19 | -46.38 |
| 4 | -49.04 | -46.43 | -46.45 | -45.48 |
| Delta | 5.14 | 0.34 | 1.84 | 1.62 |
| Rank | 1 | 4 | 2 | 3 |

**Table S6.** Analysis of Variance (ANOVA) Results for Fiber Diameter (Prepared with PVA)

| Factor | Effect | P-value |
|---|---|---|
| PVA Amount (mg) | 74.48% | 0.048 |
| Water volume in Solution (mL) | 1.32% | 0.911 |
| Range (cm) | 8.92% | 0.458 |
| Needle Diameter(G) | 7.46% | 0.515 |

* The coefficient of determination ($r^2$) for the model is 92.18%.

**Table S7.** Response Table for S/N Ratios of Bead Count Taguchi Analysis (Prepared with PVA)

| Level | PVA Amount (mg) | Water Volume in Solvent (mL) | Range (cm) | Needle Diameter (G) |
|---|---|---|---|---|
| 1 | -40.583 | 11.477 | 12.012 | 11.433 |
| 2 | -34.797 | 11.003 | 10.129 | 12.407 |
| 3 | 60 | 9.804 | 12.237 | 9.847 |
| 4 | 60 | 12.336 | 10.243 | 10.932 |
| Delta | 100.583 | 2.532 | 2.108 | 2.56 |
| Rank | 1 | 3 | 4 | 2 |

**Table S8.** Analysis of Variance (ANOVA) Results for Bead Count (Prepared with PVA)

| Factor | Effect | P-value |
|---|---|---|
| PVA Amount (mg) | 83.87% | 0.027 |
| Water volume in Solution (mL) | 3.39% | 0.663 |
| Range (cm) | 3.59% | 0.647 |
| Needle Diameter(G) | 3.38% | 0.665 |

* The coefficient of determination ($r^2$) for the model is 94.23%.

**Table S9.** Response Table for S/N Ratios of Nanofiber Diameter Taguchi Analysis (Prepared with Eudragit L100)

| Level | Eudragit L100 Amount (mg) | DMF Volume in Solvent (mL) | Range (cm) | Needle Diameter (G) |
|---|---|---|---|---|
| 1 | -39.93 | -42.3 | -41.51 | -41.93 |
| 2 | -41.34 | -42.09 | -42.09 | -42.19 |
| 3 | -42.48 | -41.84 | -42.01 | -41.91 |
| 4 | -44.11 | -41.61 | -42.24 | -41.82 |
| Delta | 4.18 | 0.69 | 0.72 | 0.37 |
| Rank | 1 | 3 | 2 | 4 |

**Table S10.** Analysis of Variance (ANOVA) Results for Fiber Diameter (Prepared with Eudragit L100)

| Factor | Effect | P-value |
|---|---|---|
| Eudragit L100 Amount (mg) | 88.53% | 0.018 |
| DMF volume in Solution (mL) | 3.61% | 0.568 |
| Range (cm) | 2.89% | 0.636 |
| Needle Diameter(G) | 0.48% | 0.95 |

* The coefficient of determination ($r^2$) for the model is 95.52%.

**Table S11.** Response Table for S/N Ratios of Bead Count Taguchi Analysis (Prepared with Eudragit L100)

| Level | Eudragit L100 Amount (mg) | DMF Volume in Solvent (mL) | Range (cm) | Needle Diameter (G) |
|---|---|---|---|---|
| 1 | -30.012 | 19.163 | -22.437 | -20.458 |
| 2 | -24.567 | -18.869 | -3.323 | 16.014 |
| 3 | -2.877 | -5.865 | -1.64 | -22.064 |
| 4 | 24.269 | -27.616 | -5.786 | -6.678 |
| Delta | 54.281 | 46.779 | 20.797 | 38.078 |
| Rank | 1 | 2 | 4 | 3 |

**Table S12.** Analysis of Variance (ANOVA) Results for Bead Count (Prepared with Eudragit L100)

| Factor | Effect | P-value |
|---|---|---|
| Eudragit L100 Amount (mg) | 55.80% | 0.012 |
| DMF volume in Solution (mL) | 32.35% | 0.026 |
| Range (cm) | 5.43% | 0.234 |
| Needle Diameter(G) | 4.26% | 0.294 |

* The coefficient of determination ($r^2$) for the model is 97.85%.

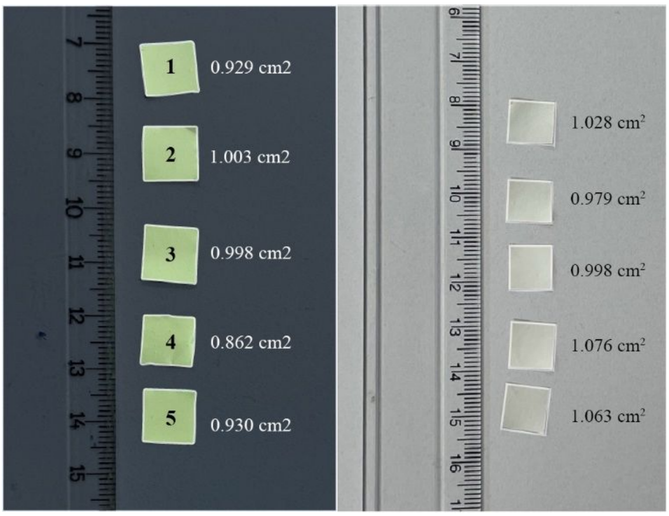


**Figure S1.** Area calculations of five randomly cut sections from PVA-based nanofibers (left) and Eudragit L100-based nanofibers (right) for active agent quantification.

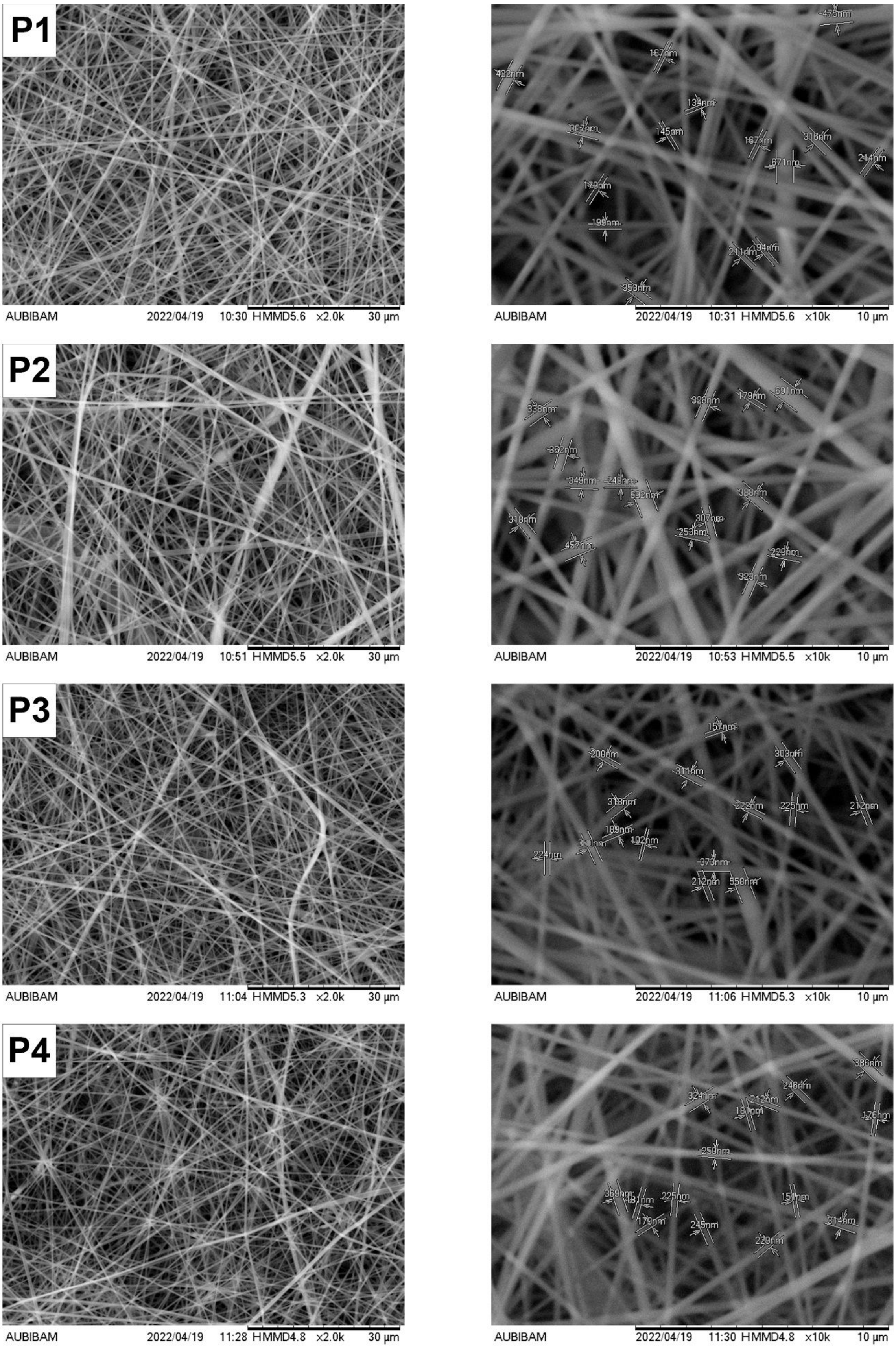


**Figure S2.** SEM images and fiber diameters of formulations P1–4, prepared according to Table 3, at 2000× magnification (left) and 10000× magnification (right).

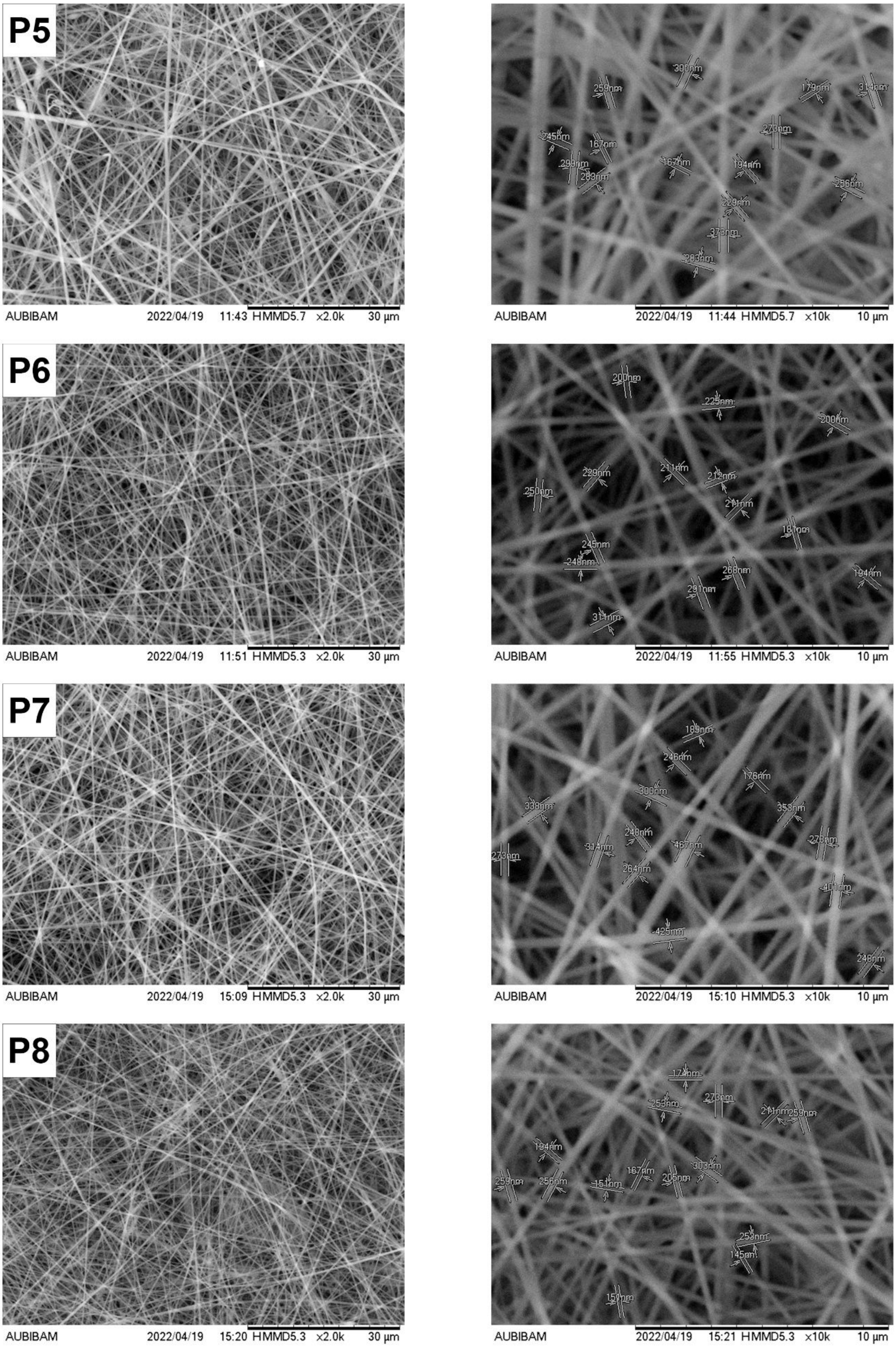


**Figure S3.** SEM images and fiber diameters of formulations P5–8, prepared according to Table 3, at 2000× magnification (left) and 10000× magnification (right).

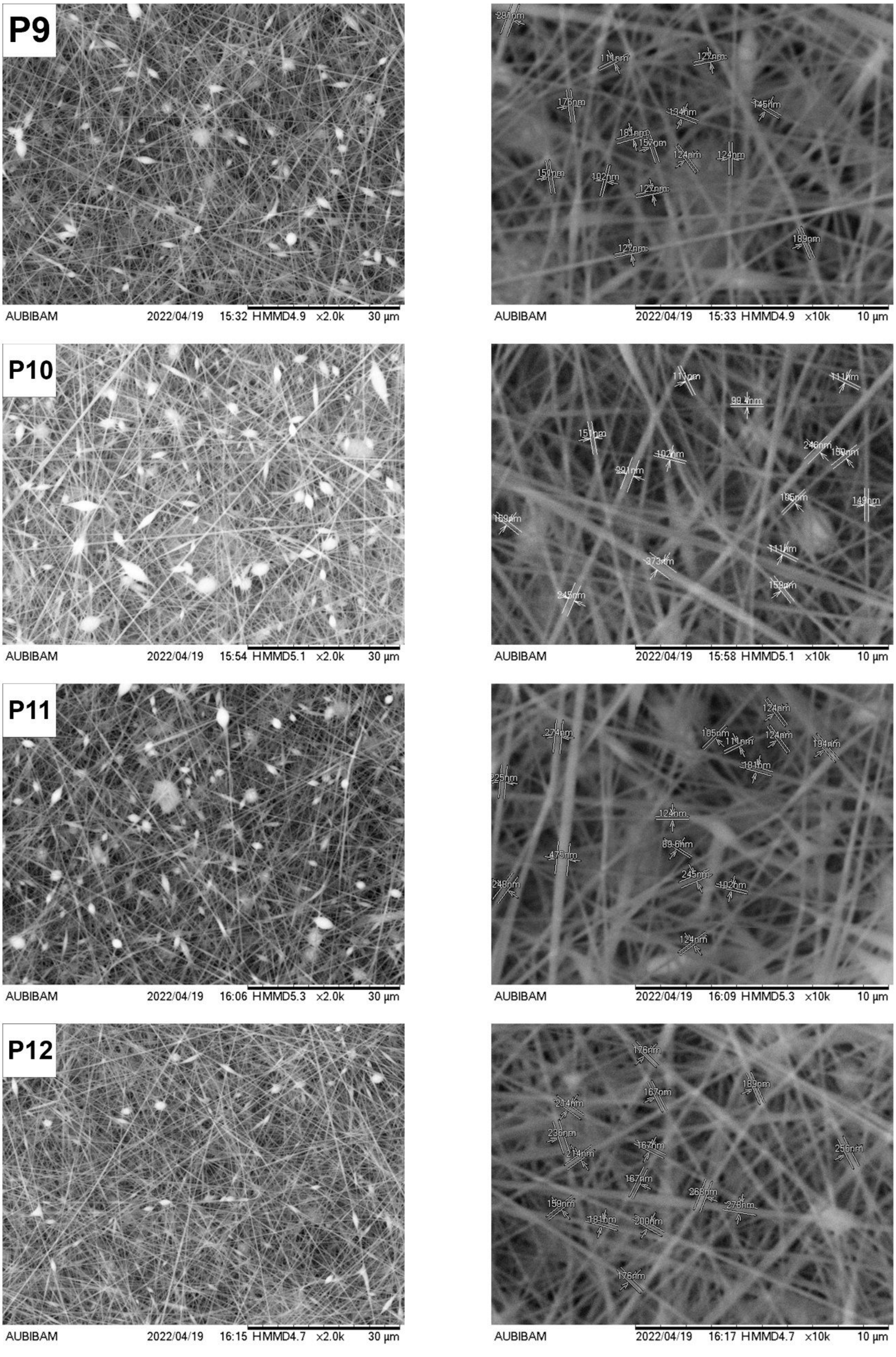


**Figure S4.** SEM images and fiber diameters of formulations P9–12, prepared according to Table 3, at 2000× magnification (left) and 10000× magnification (right).

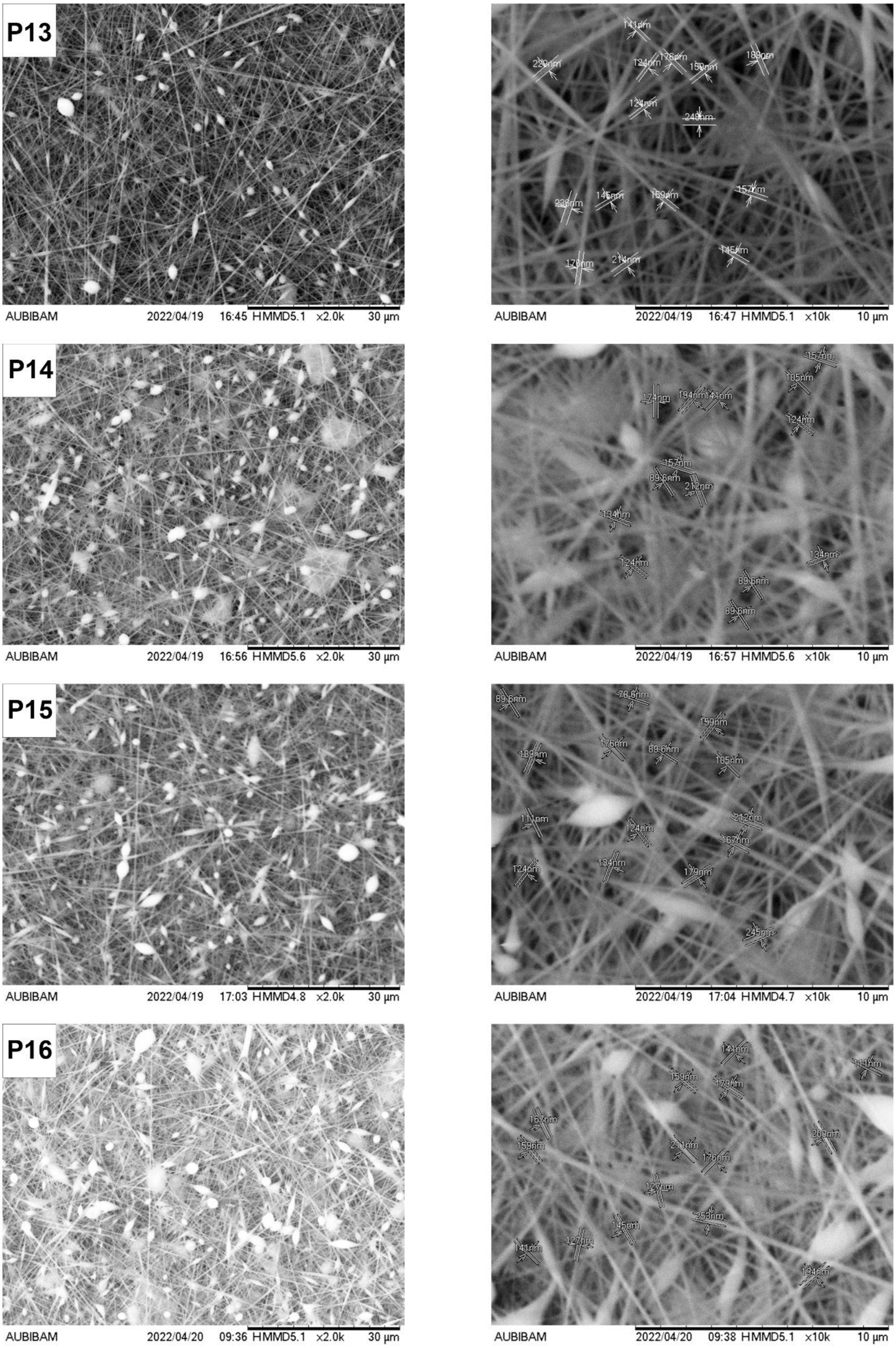


**Figure S5.** SEM images and fiber diameters of formulations P13–16, prepared according to Table 3, at 2000× magnification (left) and 10000× magnification (right).

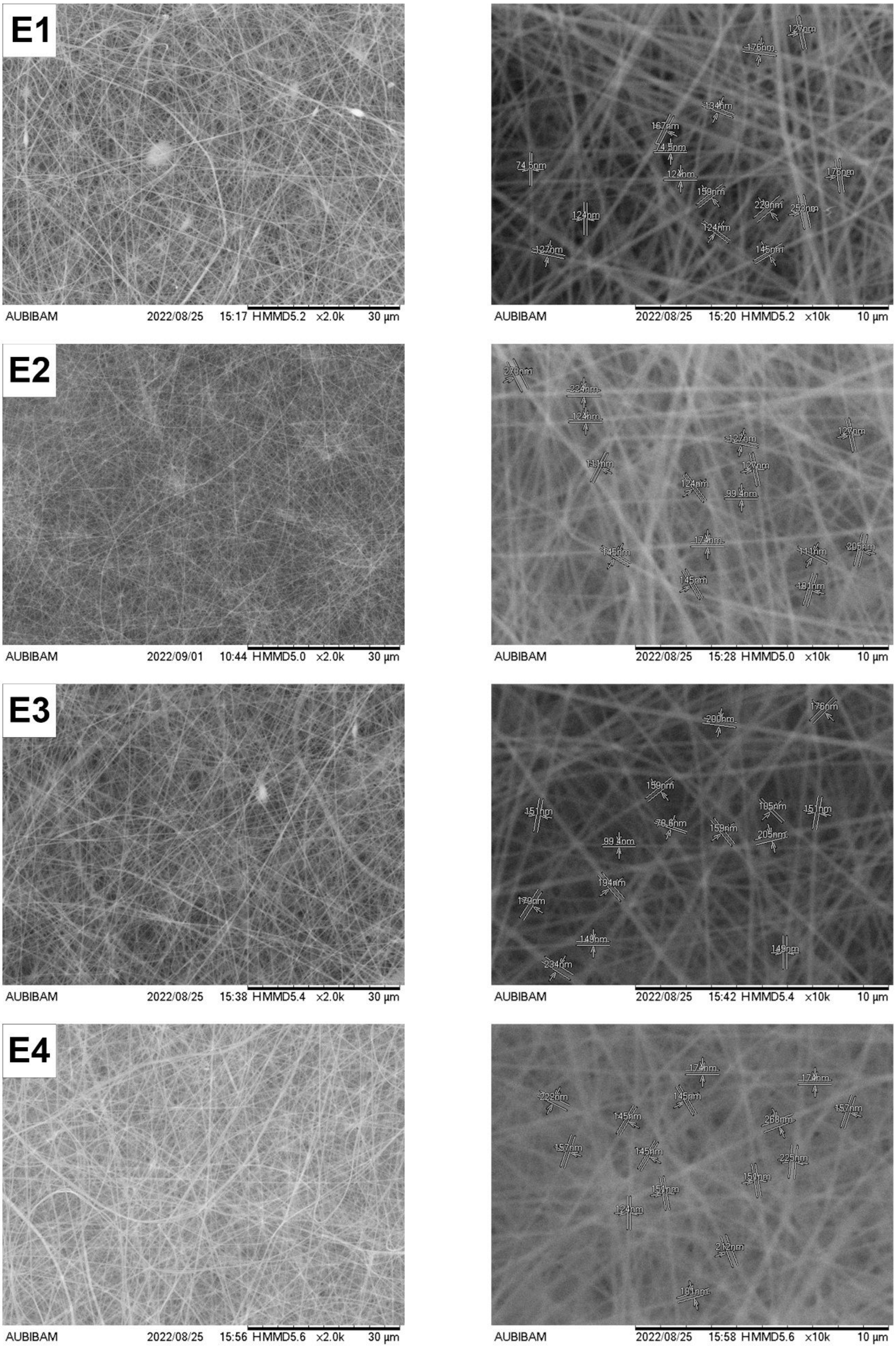


**Figure S6.** SEM images and fiber diameters of formulations E1-4, prepared according to Table 4, at 2000× magnification (left) and 10000× magnification (right).

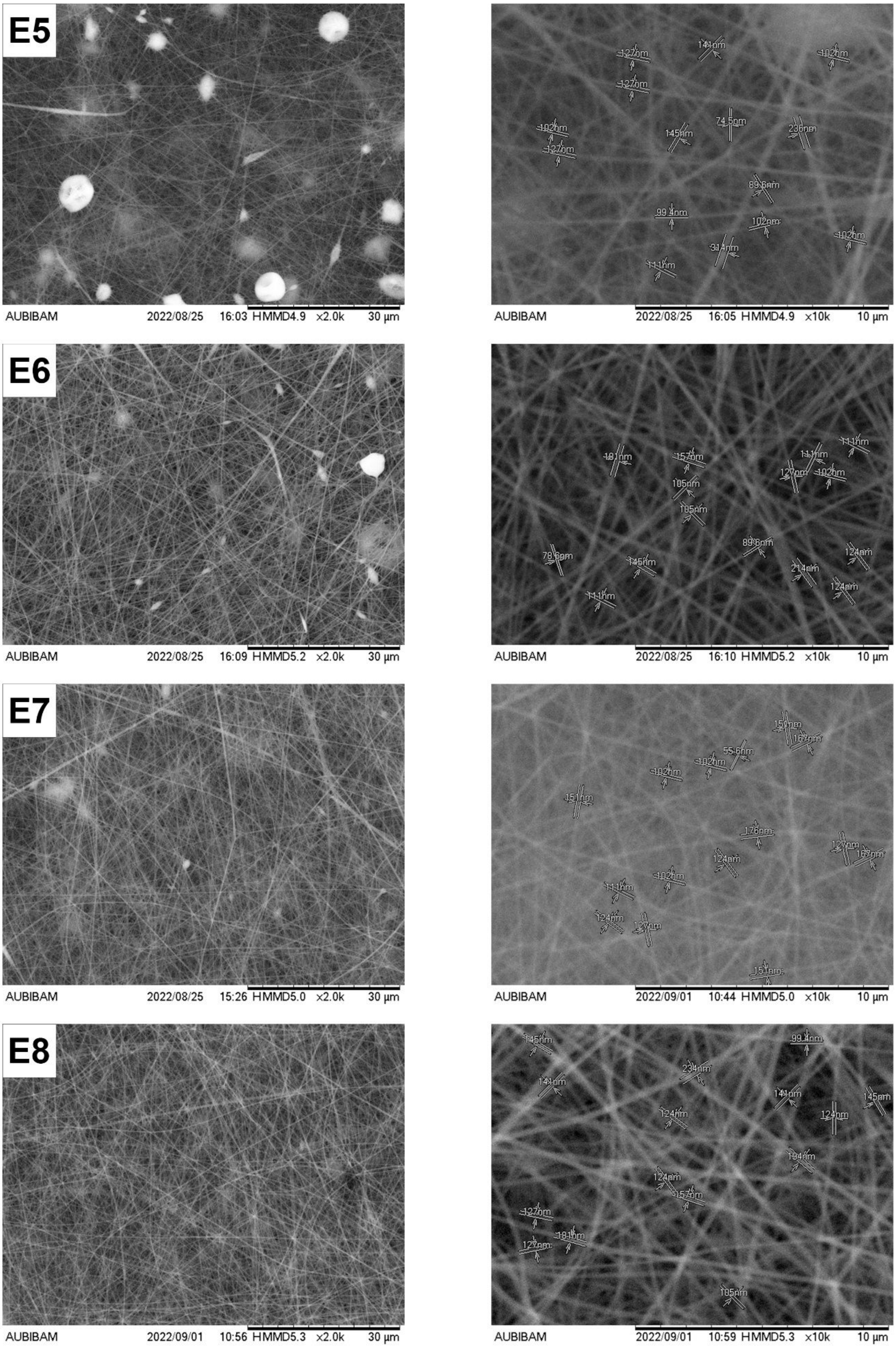


**Figure S7.** SEM images and fiber diameters of formulations E5-8, prepared according to Table 4, at 2000× magnification (left) and 10000× magnification (right).

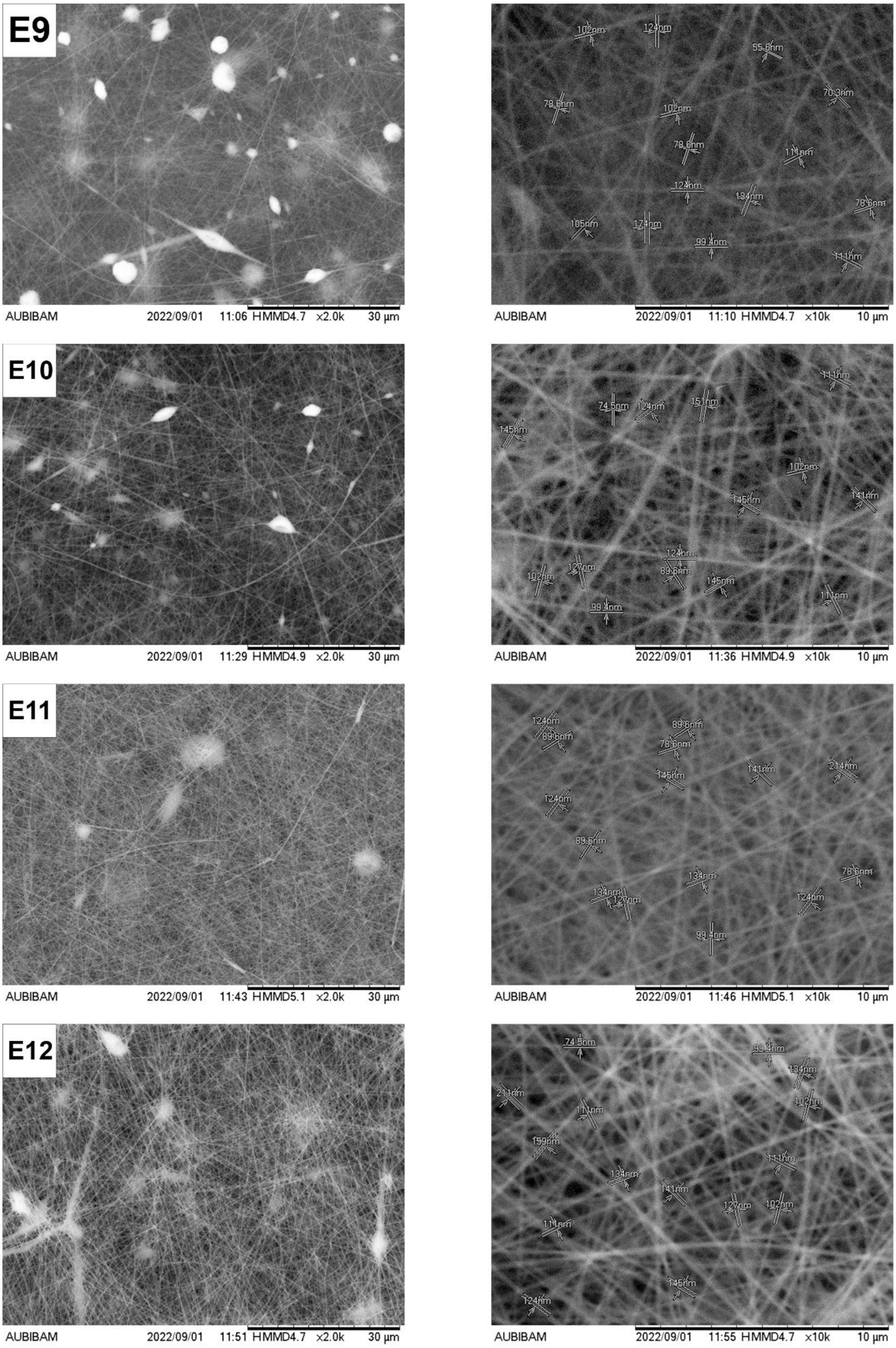


**Figure S8.** SEM images and fiber diameters of formulations E9-12, prepared according to Table 4, at 2000× magnification (left) and 10000× magnification (right).

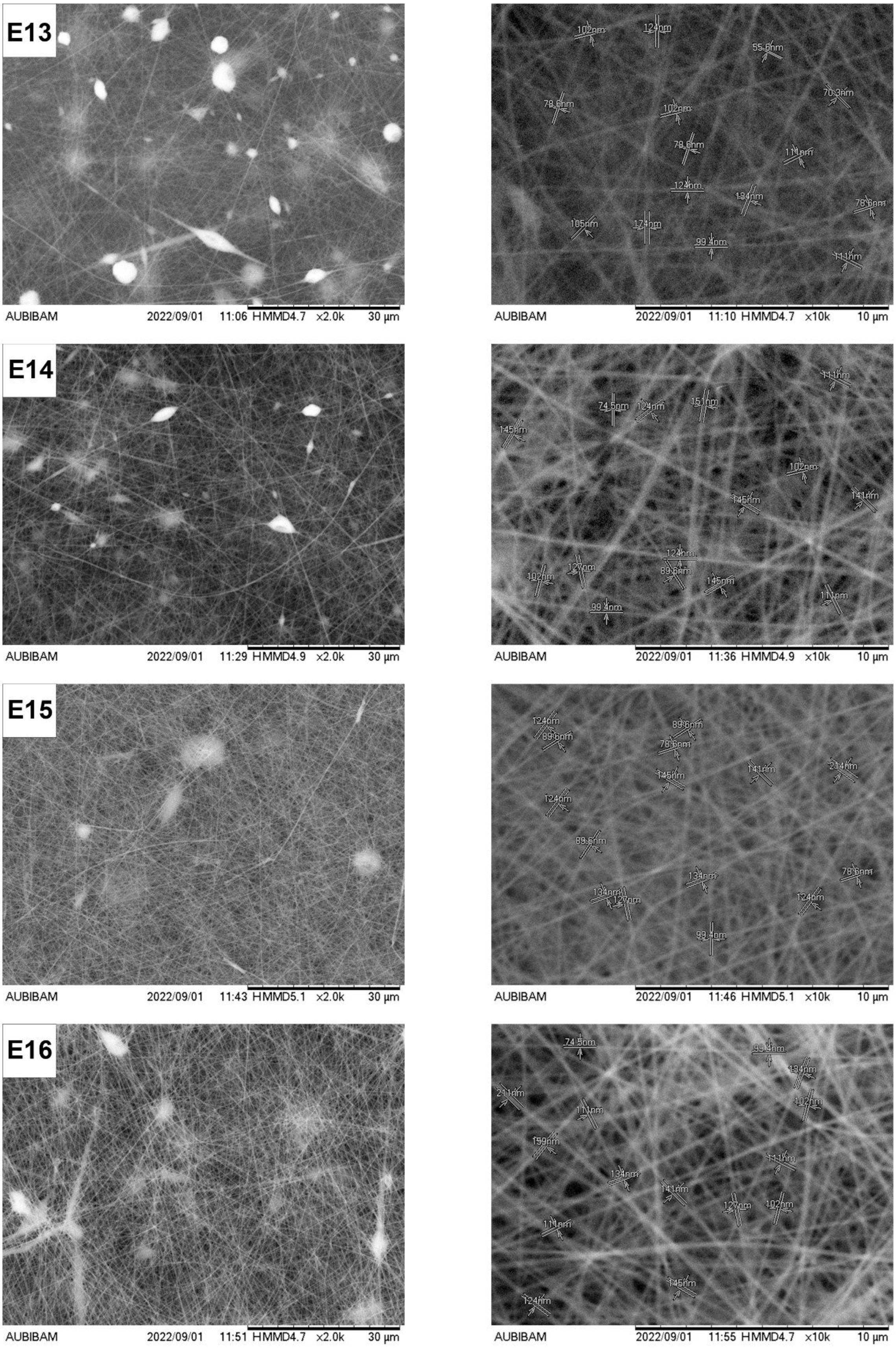


**Figure S9.** SEM images and fiber diameters of formulations E13-16, prepared according to Table 4, at 2000× magnification (left) and 10000× magnification (right).
